# Novel benzofuran/pterostilbene hybrids trigger programmed cell death and impair migration in CRC cells

**DOI:** 10.1371/journal.pone.0344602

**Published:** 2026-04-13

**Authors:** Angie Herrera-Ramírez, Rubén Becerra-Quintana, Andrés F. Yepes, Wilson Cardona-Galeano

**Affiliations:** Química de Plantas Colombianas, Faculty of Exact and Natural Sciences, Institute of Chemistry, University of Antioquia (UdeA), Medellín, Colombia; Kafrelsheikh University Faculty of Pharmacy, EGYPT

## Abstract

Colorectal cancer (CRC) remains one of the most prevalent and lethal malignancies worldwide, highlighting the urgent need for developing effective treatments. Molecular hybridization is a promising strategy for identifying new bioactive compounds. This study focused on designing and synthesizing a novel series of benzofuran-pterostilbene hybrid molecules. These compounds were successfully obtained, and their structures were elucidated by spectroscopic analysis. In addition, the activity of the hybrids was evaluated against colorectal adenocarcinoma cells. After the treatments, hybrids **6d** and **6e** exhibited the highest activity, with GI_50_ values of 11.93 ± 2.38 µM and 4.74 ± 0.38 µM, respectively, suggesting antiproliferative effects and measurable cytotoxicity under the tested conditions. Additionally, Hoechst 33342 fluorescence imaging revealed chromatin condensation and nuclear fragmentation, along with a diffuse DiOC₆ fluorescence pattern relative to the control, suggesting a form of programmed cell death, which was further supported by flow cytometric analysis showing an increased proportion of hypodiploid cells following propidium iodide staining. In parallel, wound-healing assays demonstrated impaired migration and cytotoxic effects that affected cell viability and structural integrity. Molecular docking simulations showed that compounds **6d** and **6e** bind strongly to mutant p53, CDK4, and PARP-1 proteins, which, in turn, may explain at the molecular level the *in vitro* cytotoxic effect of these compounds in SW480 colon cancer cells. Lastly, pharmacokinetic and toxicological modelling suggests that hybrids **6d** and **6e** possess optimal biopharmaceutical profiles with no major safety concerns. All these findings highlight the potential of the benzofuran-pterostilbene scaffold, with compounds **6d** and **6e** emerging as strong candidates for further evaluation against colorectal cancer.

## 1. Introduction

Colorectal cancer (CRC) is the second leading cause of cancer-related mortality worldwide, accounting for approximately 10% of all malignancies and ranking as the second most common cancer in women and the third in men. Its incidence is closely linked to modifiable lifestyle and environmental risk factors, including diet, physical inactivity, alcohol consumption, and obesity. The increasing global burden of CRC has intensified efforts to develop effective chemopreventive and therapeutic agents [1]. The rising incidence of colorectal cancer (CRC) in individuals under 50 years of age underscores the urgent need for early preventive strategies [2]. The slow progression of colorectal adenomatous polyps offers a critical window for intervention, making chemoprevention a promising approach to reduce CRC mortality [3]. Although current 5-FU–based regimens (*e.g*., FOLFIRI, FOLFIRINOX, and XELIRI) are effective, they are associated with substantial toxicity, limited tumor selectivity, and significant adverse effects [4,5]. Consequently, there is a pressing need to develop safer and more effective pharmacological agents for CRC chemoprevention.

**Fig 1:**
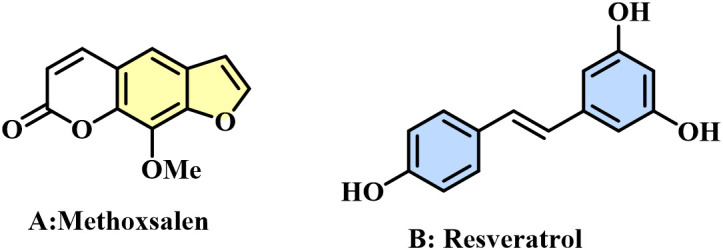
Compounds based on benzofuran and stilbenes as anticancer agents. A: Methoxsalen; B: Resveratrol.

Naturally occurring in plants, benzofuran is a heterocyclic compound that can also be synthetically produced [6]. Benzofuran derivatives are essential compounds with important biological activity and can be used to develop novel therapeutic agents with greater efficacy than current treatments. Benzofurans exhibit a broad spectrum of pharmacological properties [7–11] including anticancer activity [12–14]. Currently, the benzofuran scaffold is a key structural motif in various oncological drugs, such as methoxsalen (Oxsoralen), which is used to treat squamous cell skin cancer (Fig 1A).

Despite its frequent incorporation into bioactive molecules, the benzofuran scaffold exhibits several pharmaceutical disadvantages, particularly with respect to oral bioavailability. The lipophilic nature of the benzofuran core often facilitates extensive interactions with CYP450 enzymes, leading to rapid hepatic metabolism and a short systemic half-life. In addition, some benzofuran derivatives suffer from poor aqueous solubility, which further limits gastrointestinal absorption and favors rapid elimination [15]. A representative example is dronedarone, a benzofuran derivative that exhibits markedly lower oral bioavailability and requires higher dosing, partly due to extensive first-pass metabolism and limited absorption [16]. This example highlights how the benzofuran nucleus, while pharmacologically attractive, can impose significant challenges for drug development.

On the other hand, stilbenes are natural polyphenolic compounds with well-documented health-promoting and anticancer properties [17]. Resveratrol (Fig 1B), the most extensively studied stilbene, exhibits antiproliferative, proapoptotic, and antiangiogenic effects *in vitro* and suppresses tumor progression in multiple *in vivo* cancer models. Despite their demonstrated activity, stilbenes also present significant limitations that complicate their translation from basic research to clinical application. Resveratrol clearly illustrates this gap: although it shows strong and reproducible biological effects *in vitro*, translating these findings into human studies remains challenging due to its unfavorable pharmacokinetic profile. It is chemically unstable, susceptible to isomerization and photodegradation, exhibits low water solubility, and undergoes rapid metabolism, collectively resulting in poor oral bioavailability. As a consequence, only limited levels of the active compound reach systemic circulation, thereby reducing its therapeutic efficacy *in vivo*. [18–20]. In contrast, the development of more potent therapeutic agents has attracted increasing interest, with molecular hybridization emerging as a promising strategy. This approach involves the chemical integration of two or more bioactive moieties into a single hybrid molecule, aiming to enhance pharmacological efficacy, optimize pharmacokinetic properties, and enable simultaneous modulation of multiple biological targets [21–25]. Hybridization has been associated with improved anticancer activity and selectivity, as well as reduced adverse effects [26], highlighting the anticancer properties of reported Benzofuran- and stilbene-based hybrids [27–29]. Notable examples of clinically relevant benzofuran-based hybrids include fruquintinib, a benzofuran–pyrimidine hybrid approved for metastatic colorectal cancer [30,31]; BNC105, a benzofuran–combretastatin A-4 hybrid currently under phase I/II clinical evaluation for metastatic renal and ovarian cancers [32]; and abexinostat, a benzofuran–vorinostat hybrid approved for refractory follicular lymphoma [33–35]. In addition, several isatin–benzofuran hybrid scaffolds have demonstrated potent anticancer activity against colorectal cancer models, including N-acylhydrazone-linked derivatives showing promising efficacy in HT29 and SW620 cell lines [27,36].

The resveratrol-based hybrid RIH (resveratrol–isonicotinoyl hydrazone) exhibited notable cytotoxic activity against SW480 (19.0 µM) and SW620 (38.4 µM) colorectal cancer cell lines, showing improved activity and selectivity compared to the reference drug 5-fluorouracil (5-FU) and PIH (pyridoxal isonicotinoyl hydrazone) [37]. Similarly, one compound previously reported by our group exhibited promising antiproliferative activity against SW480 and SW620 cells, with IC_50_ values in the low micromolar range and selectivity indices greater than 1 after 48 h, surpassing those of resveratrol, 5-fluorouracil, and an equimolar curcumin–resveratrol mixture [38]. Besides, a series of stilbene–coumarin hybrids showed strong and selective cytotoxicity against SW480 cells, identifying two compounds with low micromolar IC_50_ values (1.01 and 6.92 µM), high selectivity indices (67.8 and >400), and sustained activity over time, outperforming both parent compounds (resveratrol and coumarin) as well as the reference drug (5-FU) [39].

### Principio del formulario

#### Final del formulario.

**Fig 2:**
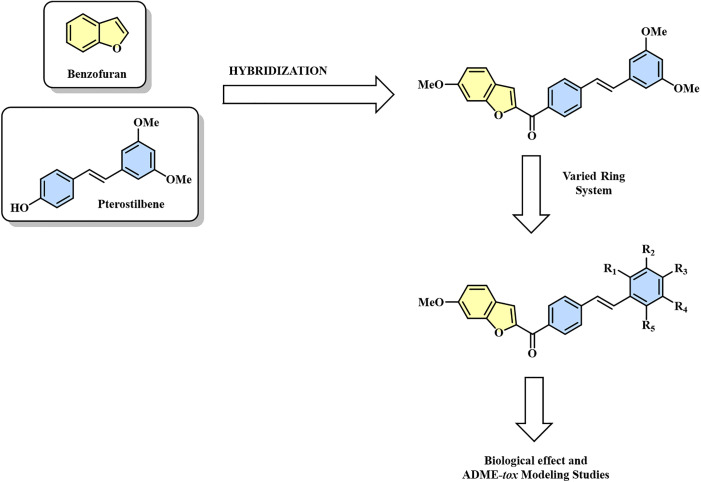
Designing benzofuran-pterostilbene hybrids as anticancer agents.

Given the aforementioned background and the urgent need to develop innovative therapeutic alternatives for the treatment of colorectal cancer, we used the Mizoroki-Heck cross-coupling reaction as a key step in the synthesis of hybrids that fused the benzofuran ring to the pterostilbene framework (Fig 2). Additionally, we assessed their biological activity in colon adenocarcinoma cells (SW480) to determine their chemopreventive potential against this cancer type. The newly designed molecules aim to preserve the beneficial biological features associated with the benzofuran and stilbene frameworks while enhancing chemical stability and bioavailability, thereby addressing key limitations that restrict their use.

## 2. Results and discussion

### 2.1. Chemistry

**Scheme 1:**
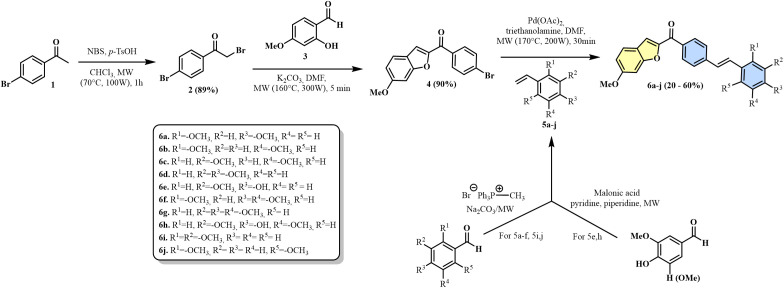
Synthesis of benzofuran-pterostilbene hybrids 6a-j.

The first step in the synthesis of the hybrids was to prepare α-bromoketone **2** by brominating 4-bromoacetotophenone (**1**) and NBS with the use of microwave irradiation (yield 89%) [40].The condensation of compound **2** with 2-hydroxy-4-methoxybenzaldehyde **3** in basic medium microwave assisted gave the (4-bromophenyl)(6-methoxybenzofuran-2-yl)methanone **4** (yield 90%) [41]. Finally, a cross-coupling reaction between this compound and various styrenes (**5**) under palladium catalysis [42], leading to the formation of hybrids **6a-6h** with 20%−60% yields. Compound **6j** was obtained as a mixture of isomers (*E*,*Z* ratio 4:1). Microwave-assisted Wittig reactions between substituted aldehydes and methyltriphenylphosphonium bromide [39,42,43] and Knoevenagel condensation reactions between 4-hydroxybenzaldehydes with malonic acid were used to create styrene (**5**) [39,42,44] (Scheme 1).

All hybrids were characterized using high-resolution mass spectrometry and proton and carbon-13 nuclear magnetic resonance spectra. HRMS-ESI spectra (m/z) showed characteristic [M + H]^+^ peaks that corresponded to the hybrid molecular weights. All of the signals have been assigned to specific H or C atoms using standard δ-values and *J*-constants. The ^1^H NMR spectra of the hybrids dissolved in CDCl_3_ showed signals belonging to the benzofuran ring around 7.68 ppm or 7.70 ppm. The signal corresponding to the hydrogens of stilbene is around 7.20 ppm and 7.10 ppm as a doublet with *J* ~ 16 ppm. ^13^C-NMR spectra showed characteristic signals at ~183 ppm corresponding to the C = O groups and at 116.9 ppm due to the presence of -O-C = **C**H- (benzofuran).

### 2.2. *In vitro* biological activity

#### 2.2.1. Preliminary screening.

The development of new therapeutic agents targeting colorectal cancer (CRC) remains a critical priority due to the high incidence and mortality rates associated with this malignancy. In this study, we designed and synthesized a library of benzofuran derivatives and evaluated their biological activity *in vitro*. All compounds were screened at an initial concentration of 100 µM to assess their cytotoxic or cytostatic potential against SW480 human colorectal adenocarcinoma cells, a standard 2D model for colorectal cancer research. Additionally, hybrids **6e** and **6d** were selected for further *in vitro* evaluation through a seven-dose assay. The results of their activity are presented separately under two subsections, corresponding to the one-dose and seven-dose analyses.

#### 2.2.2. *In vitro* activity at one-dose screening.

**Fig 3:**
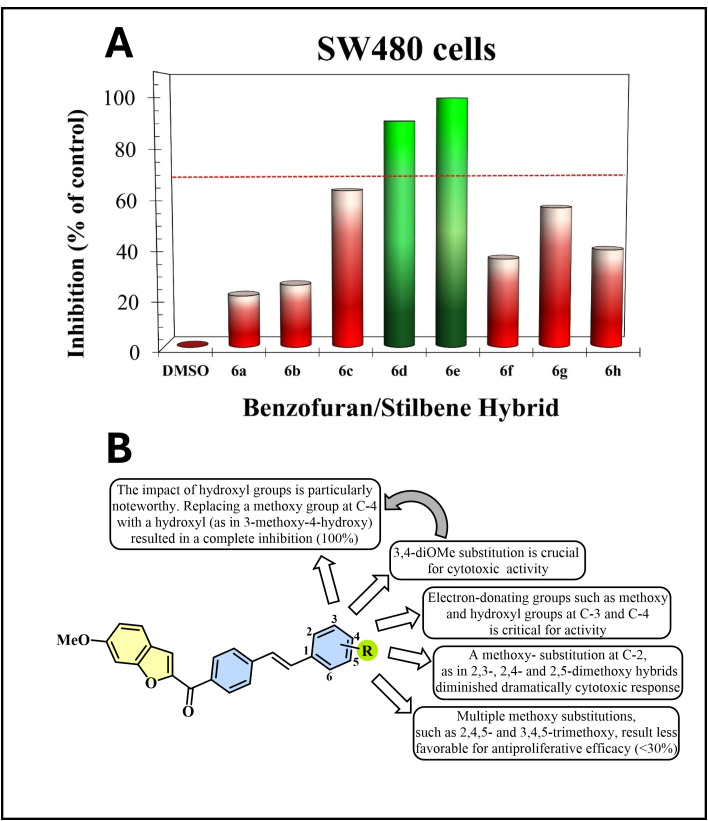
Inhibition (%) values and SAR analysis of the Benzofuran/Stilbene hybrid compounds. A) A bar graph depicting the inhibition (%) values of the synthesized Benzofuran/Stilbene hybrid compounds (6a-i). Green bars represent the most active hybrids, and red bars correspond to non-active molecules. The dotted red line denotes the minimum activity threshold. DMSO-treated cells were used as the baseline for calculating inhibition percentages, with 100% viability (zero percent inhibition) assumed for this control group. B) Structure-Activity Relationship (SAR) analysis illustrating the cytotoxic effects of the hybrid compounds against human SW480 cells.

The one-dose results for each hybrid were expressed as the percentage growth of treated cells, as summarized in **Table 1**. Here, we included inhibition and lethality percentages, providing preliminary insights into their effects on cell viability and proliferation. Based on inhibition levels, compounds were classified into three activity groups: low (≤30%), moderate (30–70%), and high (≥70%). According to the results, most of the compounds evaluated exhibited limited inhibitory activity. Thus, compounds **6a** and **6b** displayed the lowest activity with inhibition percentages of 20.87 and 25.25, respectively, followed by hybrids **6c**, **6f-h,** which exhibited moderate activity but did not reach the threshold (70%) required for progression to subsequent assays. In addition, it was observed that hybrids **6d** and **6e** exhibited the greatest biological activity, with a pronounced antiproliferative activity, as demonstrated by inhibition percentages nearing 100% (Fig 3A). Furthermore, compound **6e** caused a slight reduction in cell viability relative to the initial seeding density, suggesting this compound may display cytostatic and cytotoxic effects under the conditions evaluated.

**Table 1:** One-dose screening in malignant cells (SW480).

Benzofuran/Stilbene hybrids		
Hybrid	Inhibition (%) ± SD^a^	Lethality (%) ± SD
6a	20.87 ± 4.02	N.L.
6b	25.25 ± 9.46	N.L.
6c	63.28 ± 3.93	N.L.
**6d**	**90.84 ± 2.34**	**N.L.**
**6e**	**100.00 ± 3.78**	**17.06 ± 3.78**
6f	35.82 ± 5.49	N.L.
6g	56.40 ± 5.09	N.L.
6h	39.47 ± 3.14	N.L.
6i	N.I.	N.L.

The results from the one-dose screening at 100 μM are presented as the mean percentage increase in treated cell growth. The most active compounds are indicated in bold. Values are expressed as the mean ± standard deviation (SD). N.L. (Non-Lethal); N.I. (No Inhibition).

Similar results have been reported by other authors. Wagdy and colleagues presented a series of benzofuran–isatin hybrids evaluated through the Developmental Therapeutics Program at the National Cancer Institute (NCI), USA. Seven selected conjugates were submitted for preliminary *in vitro* evaluation against a panel of 55 human cancer cell lines, covering a broad spectrum of tumor types. Most of the tested compounds exhibited minimal to moderate inhibitory activity, except for one compound, which demonstrated excellent antiproliferative activity against nearly all cancer cell lines (mean growth inhibition of 87.33%). Furthermore, the active compound induced a lethal cytotoxic effect (Growth inhibition >100%) in several cell lines, including colorectal cancer [27]. Likewise, we previously reported a series of 5-FU/pterostilbene with similar activity in terms of inhibition percentage at one single dose screening, finding two compounds that emerged as notably active candidates within the series with GI_50_ values of 35.93 ± 5.07 µM and 34.90 ± 2.27 µM for hybrids 5e and 5g, respectively, each demonstrating substantial antiproliferative effects by achieving growth inhibition percentages greater than 70% in the *in vitro* screening assays [45]. These findings suggest that benzofuran-linked pterostilbene may have strong potential for further development as a lead structure in anticancer drug discovery.

#### 2.2.3. Structure–Activity Relationship study (SAR).

Considerable differences in inhibitory activity among hybrids **6a-i** were observed based on their substitution patterns, as depicted in the bar graph (Fig 3A). These findings informed the subsequent structure–activity relationship (SAR) analysis presented in Fig 3B. 1) Substitution at C-3 and C-4 positions significantly enhanced antiproliferative activity. Compounds bearing substituents at positions 3,4-dimethoxy and 3-methoxy-4-hydroxy- exhibited the highest levels of inhibition (90.84% and 100%, respectively). This suggests that the presence of electron-donating substituents, such as methoxy and hydroxyl, at these positions is critical for activity. 2) In contrast, substitutions involving the C-2 position, such as those in the 2,3-, 2,4-, and 2,5-dimethoxy analogs, were associated with comparatively low levels of inhibition (<30%). This observation implies that substitution at C-2 resulted in less favorable antiproliferative efficacy or may negatively modulate the molecule’s interaction with its biological target. 3) The impact of hydroxyl groups is particularly noteworthy. Replacing a methoxy group at C-4 with a hydroxyl (as in 3-methoxy-4-hydroxy) resulted not only in a complete growth inhibition (~100%) but also a substantial lethality grade of around 17.06%, suggesting a potentiating effect of the hydroxyl group, possibly due to enhanced hydrogen bonding or improved solubility. 4) Molecules with multiple methoxy substitutions, such as 2,4,5- and 3,4,5-trimethoxy, displayed moderate activity (35.82% and 56.40%, respectively). These results indicate that increasing the number of methoxy groups does not necessarily translate into higher activity, underscoring the importance of their positional arrangement rather than their quantity.

#### 2.2.4. *In vitro* activity at a seven-dose assay.

**Fig 4:**
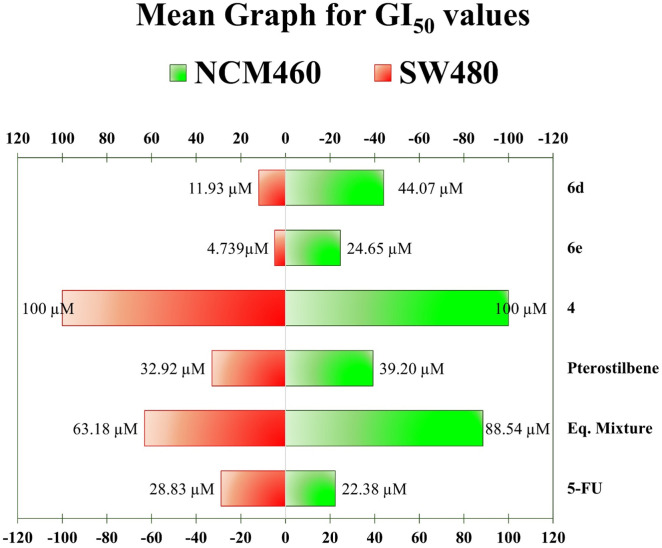
Mean graph for GI_50_ values for the most active hybrids 6d and 6e. Green right bars represent values against non-malignant NCM460 cells. Red left bars represent values over malignant SW480 cells. A shorter red bar combined with a larger green bar indicates enhanced cytotoxicity toward cancer cells and improved selectivity.

Achieving selective cytotoxicity (eliminating cancer cells while preserving the viability of normal cells) is a fundamental goal in developing effective and safe anticancer therapies. Because of this, we evaluated the most active benzofuran/pterostilbene hybrids, **6d** and **6e** (those that satisfied the predetermined threshold inhibition at one dose), against SW480 and NCM460 cells using seven-dose serial dilutions (ranging from 0.10 µM to 100 µM). The dose-response curves allowed the determination of critical parameters, including GI_50_ (the concentration required to inhibit cell growth by 50%), TGI (the concentration at which total growth inhibition occurs), and LC_50_ (the concentration at which the cell population is reduced by 50% compared to the initial time point), as seen in **Table 2**. The results showed that the hybrid compounds exhibited markedly enhanced antiproliferative activity compared to their parent structures, as reflected by lower GI_50_ values. This improvement underscores the effectiveness of molecular hybridization strategies in increasing cytotoxic potency, as both compounds were more active than the parental compound **4** (Benzofuran) and pterostilbene. In addition, it is important to notice that hybrids **6d** and **6e** demonstrated nearly five- and seven-fold higher selectivity, respectively, indicating a substantial improvement in targeting efficacy compared to the reference drug 5-FU as evidenced by higher GI_50_ values in non-malignant NCM460 cells (Fig 4), which is particularly important given the fundamental goal of developing effective and selective anticancer therapies.

**Table 2:** Seven-dose screening on SW480 and NCM460 cells.

Hybrid Compound	SW480			NCM460			SI
	GI_50_	TGI		LC_50_	GI_50_	TGI	LC_50_
	(µM ± SD)	(µM ± SD)		(µM ± SD)	(µM ± SD)	(µM ± SD)	(µM ± SD)
**6d**	11.93 ± 2.38****	>100	>100	44.07 ± 5.99	>100	>100	3.69****
**6e**	4.74 ± 0.38****	>100	>100	24.65 ± 1.24	>100	>100	5.20****
**4 (Benzofuran)**	>100	>100	>100	>100	>100	>100	N.C.
**Pterostilbene**	32.92 ± 1.46	60.78 ± 1.58	90.02 ± 2.84	39.20 ± 3.30	61.61 ± 16.74	96.55 ± 2.50	1.19
**Equimolar Mixture**	63.18 ± 3.59	>100	>100	88.54 ± 1.54	>100	>100	1.40
**5-FU**	28.83 ± 3.61	>100	>100	22.38 ± 2.30	>100	>100	0.78

Cytotoxic effects 48 hours post-treatment. Dose–response curves were subsequently used to calculate GI_50_, TGI, and LC_50_ values. The results are reported as the mean ± standard deviation (SD) based on data from independent technical replicates. To assess selectivity, the GI_50_ value in normal NCM460 cells was divided by the GI_50_ value in cancerous SW480 cells. The study included 5-fluorouracil (5-FU) as a standard chemotherapy control, with Pterostilbene and compound **4 (Benzofuran)** as reference molecules. NC = Not calculated. ****p≤0.0001.

Other authors have reported comparable findings. Wagdy and colleagues reported a benzofuran–isatin hybrid that exhibited significant activity across multiple cancer cell lines, with GI_50_ values ranging from 1.92 to 3.18 µM across subpanels. Notably, the colon and renal cancer subpanels emerged as the most susceptible, exhibiting mean GI_50_ values of 1.92 µM and 1.98 µM, respectively. In addition, they reported a further investigation beyond the NCI panel, showing that compound **5d** significantly reduced the viability of colorectal cancer cells, demonstrating approximately 52% inhibition in SW620 cells. The compound also showed potent dose-dependent cytotoxicity, with IC_50_ values of 9.8 µM and 6.5 µM in SW620 and HT-29 cells, respectively. While it was slightly less powerful than the reference drug Irinotecan in SW620 cells, compound **5d** displayed comparable efficacy in HT29 cells, suggesting its potential as a promising lead structure for further development in colorectal cancer therapy. Importantly, this research agrees with our findings, as compound **5d** exerted a non-significant effect on normal fibroblast cells, underscoring its selective mechanism of action and reinforcing its potential as a promising lead candidate for further development in colorectal cancer therapy [27]. Likewise, Wawszczyk et al. reported that pterostilbene inhibits proliferation and induces cell death in HT29 colon cancer cells. Depending on the concentration, it also decreased cell growth, causing cell cycle arrest at the G1 phase, and/or triggered apoptosis mediated by downregulation of the STAT3 and AKT signaling pathways, which are key for cell survival and proliferation [46]. These findings support our results and thus reinforce the potential of benzofuran/pterostilbene hybrids as candidates for developing new therapeutic alternatives against colorectal cancer.

### 2.3. Approaching cellular integrity and dynamics of hybrids 6d and 6e

#### 2.3.1. Optical microscopy evaluation.

**Fig 5:**
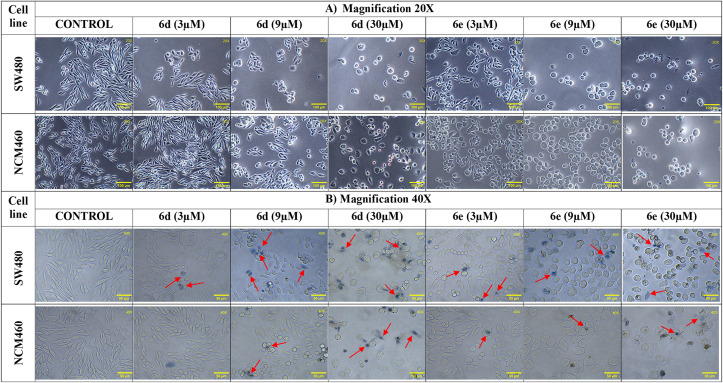
Optical microscope images. Cell viability of SW480 and NCM460 cells after treatment with hybrids **6d** and **6e**. Cells were stained with trypan blue dye. **A)** Magnification 20x; **B)** Magnification 40x. Red arrows show dead cells stained with trypan blue dye.

As illustrated in **Fig 5**, the optical microscopy analysis revealed profound alterations in SW480 colorectal adenocarcinoma cells following treatment with hybrids **6d** and **6e**. Treated cells displayed evident reduction in the number of cells; moreover, the remaining ones exhibited hallmark indicators of cell death, including changes in size and shape, membrane blebbing, and nuclear fragmentation. These features are widely recognized as classical hallmarks of apoptosis and suggest that the hybrids may induce a programmed cell death mechanism [47]. Additionally, cytoplasmic vacuolization and detachment from the substrate indicate a breakdown in cell–substrate adhesion and increased cellular stress, potentially linked to cytoskeletal disruption. These events often result in regulated necrotic cell death, including paraptosis, methuosis, or pyroptosis. Additionally, depending on the combined stimuli or the surrounding cellular environment, a moderate level of apoptosis or autophagy-associated cell death may also occur [48].

Trypan blue staining has been successfully used to assess membrane permeability, where positive staining indicates compromised lipid bilayer integrity [49]. Given the observed detachment and blebbing in SW480 cells, plasma membrane damage likely contributes to the cytotoxic effects of the hybrids. The fewer morphological changes in the non-malignant NCM460 cell line under identical treatment conditions (at low concentrations near the GI_50_) highlight the selective activity of these compounds. Selectivity indices derived from dose–response assays support this differential behavior and suggest a promising therapeutic candidate. This is particularly relevant, as minimizing off-target cytotoxicity remains a challenge in anticancer drug development. It has been documented that nuclear fragmentation suggests potential induction of intrinsic apoptotic pathways, possibly through mitochondrial involvement and caspase activation [50]. However, further studies are warranted to confirm this mechanism.

Taken together, the morphological data, combined with the differential responses in malignant and non-malignant cells, suggest that hybrids **6d** and **6e** could be important candidates for designing more selective agents for colorectal cancer treatment.

#### 2.3.2. Fluorescence microscopy with DiOC₆.

**Fig 6:**
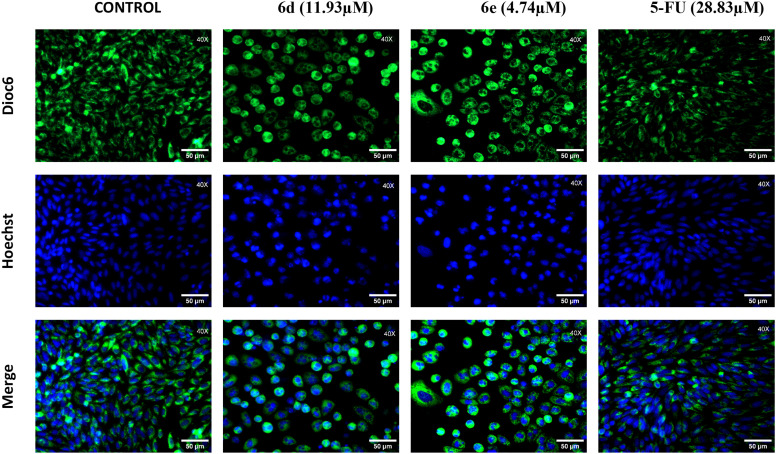
Fluorescence microscope images. SW480 cells after treatment with the GI_50_ concentration of hybrids **6d**, **6e,** and the reference drug 5-FU. Cells were stained with DIOC6 and Hoechst. Magnification 40x.

Mitochondria are essential organelles in eukaryotic cells, responsible not only for ATP production but also for regulating ion homeostasis, redox balance, and various forms of cell death [51,52]. A central parameter of mitochondrial function is the mitochondrial membrane potential (ΔΨm), which reflects the electrochemical gradient across the inner mitochondrial membrane and is crucial for maintaining mitochondrial activity and cellular viability. Loss of ΔΨm is a hallmark of mitochondrial dysfunction and is commonly observed in early stages of apoptosis and other regulated cell death mechanisms. Detecting changes in ΔΨm provides critical insight into mitochondrial health and cellular responses to stress or toxic compounds [53,54]. On the other hand, the plasma membrane potential (PMP), plays a vital role in maintaining cellular function. Emerging evidence indicates that a dynamic membrane potential is critical for many processes, including cell cycle and cell volume control, proliferation, muscle contraction, and wound healing. Modulation of the PMP is therefore a potential target for new therapeutic approaches, ranging from cancer treatment to stem cell therapies and regenerative medicine [55,56]. Considering this, we employed a green-fluorescent, cationic, and lipophilic dye DiOC6 (3,3’-dihexyloxacarbocyanine iodide) to evaluate the ability of compounds **6d** and **6e** to induce depolarization of the plasma membrane or changes in Δ*Ψm*. Under depolarizing conditions, such as exposure to pro-apoptotic agents, DiOC6 redistribution results in a marked reduction in fluorescence, detectable by fluorescence microscopy. As illustrated in **Fig 6**, we found that SW480 cells treated with the GI_50_ concentration of the hybrids exhibited a diffuse DiOC6 fluorescence pattern compared to the control. This observation suggests disruption of the mitochondrial membrane potential or plasma membrane depolarization, both critical events that compromise mitochondrial integrity and can initiate regulated cell death pathways. These changes have also been linked to cell cycle arrest, which, although generally considered a protective response to cellular stress, can be advantageous in cancer treatment by preventing the proliferation of malignant cells and enhancing the cytotoxic activity of anticancer compounds [57]. In addition, we used Hoechst 33342, a widely used cell-permeable fluorescent dye that selectively binds to the minor groove of double-stranded DNA, with a preference for A-T-rich regions, enabling high-resolution nuclear staining in both live and fixed cells. Its compatibility with live-cell imaging, minimal cytotoxicity at optimized concentrations, and strong blue fluorescence emission make it particularly well suited for dynamic monitoring of nuclear changes. In our experimental results, Hoechst 33342 allowed clear visualization of nuclear morphology, revealing features such as chromatin condensation and nuclear fragmentation. These structural alterations are well-established hallmarks of apoptosis, supporting the possible involvement of this type of cell death in response to the treatment with the hybrid molecules, which were not evidenced with the reference drug. Even when the predominant mechanism seems to be apoptosis, other forms of programmed cell death, such as parthanatos or ferroptosis, cannot be entirely excluded without additional markers. Moreover, the observation of large multinucleated cells with shared cytoplasm suggests that molecules **6d** and **6e** may induce mitotic catastrophe, supporting the hypothesis that the mechanism involves a hybrid mechanism in which mitotic catastrophe initiates the process and subsequently induces apoptotic execution.

#### 2.3.3. Cell cycle distribution.

**Fig 7:**
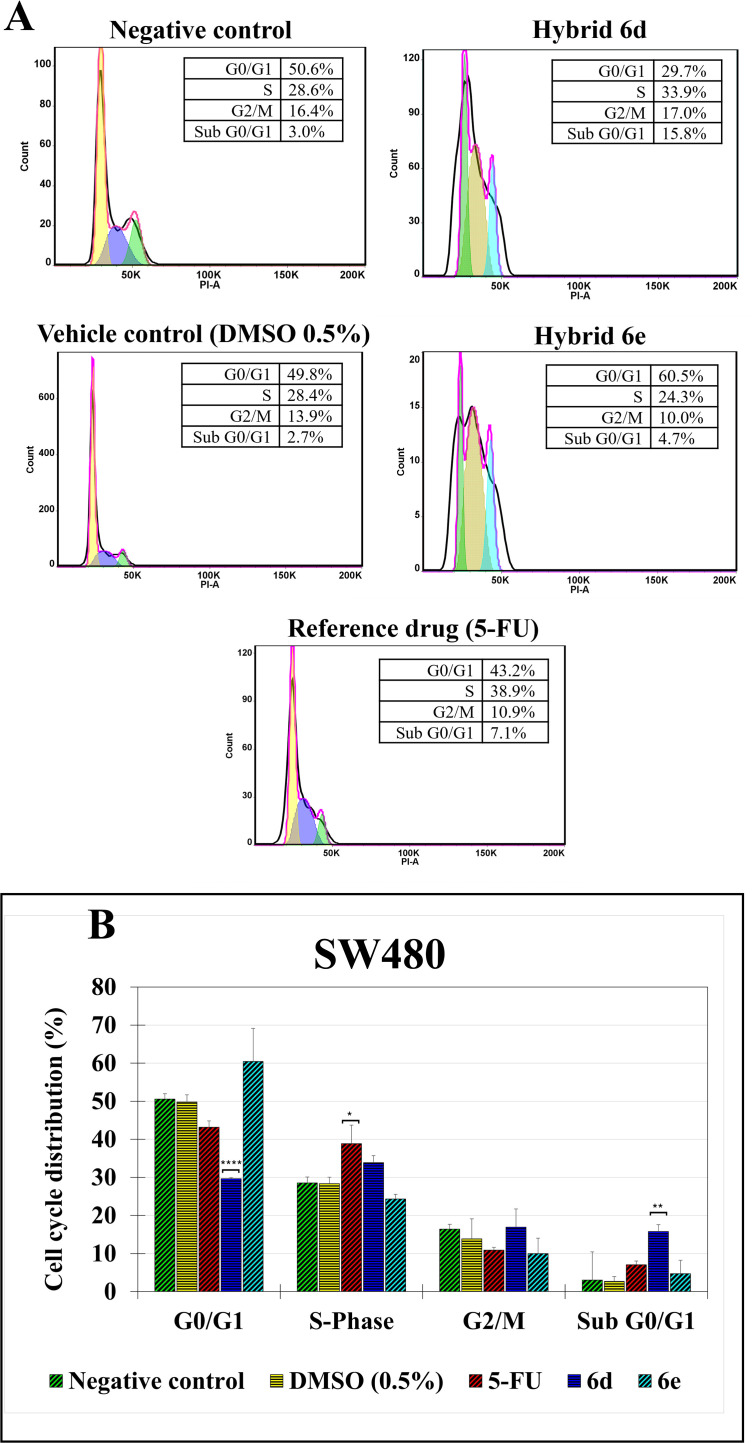
Propidium iodide (PI)–based analysis of DNA content after the treatment with hybrids 6d and 6e. **A)** The graphs show the percentage of cells in G0/G1, S, and G2/M phases. Negative control: cells without any treatment; Vehicle control: cells treated with the solvent DMSO. 5-FU was used as the reference drug. **B)** Data presentation using a bar chart. (*p < 0.05; **p < 0.01; **** *p* < 0.0001).

Cell cycle analysis has become an important technique for characterizing how cell populations progress through the cell cycle phases and for detecting perturbations, such as cell cycle arrest or altered proliferation kinetics, following treatment with various agents. Using methods such as DNA content staining with propidium iodide followed by flow cytometry, it is possible to quantify the fraction of cells in the different phases, thereby revealing population-level cell cycle distribution [58]. In the present research job, SW480 cells were treated with hybrids 6d and 6e to detect such changes. According to the results, we evidenced that fluorescence microscopy revealed chromatin condensation and nuclear fragmentation, morphological alterations commonly associated with ongoing cell death processes [59], and, consistent with these observations, the flow cytometry analysis showed that treatment with compound 6d produced a marked increase in the sub-G0/G1 population in the DNA content analysis (Fig 7A). Cells in this region exhibit hypodiploid DNA content, reflecting fragmented or degraded DNA that is generally indicative of cell death–associated DNA loss. In parallel, a reduction in the G0/G1 population was detected after exposure to 6d, suggesting that a portion of cells originally in this phase may have transitioned into a state of DNA degradation. Together, these changes indicate that this hybrid induces significant cytotoxic effects (Fig 7B), accompanied by chromatin alterations and a reduction in DNA content. In contrast, compound 6e produced only slight increases in both G0/G1 and sub-G0/G1, and these variations were not statistically significant. This pattern suggests it exerts only a limited impact on cell integrity or cell-cycle progression, without generating substantial DNA loss or detectable shifts in distribution under the tested conditions. Similarly, compound 6d induced a slight, non-significant accumulation in S phase, indicating only a weak tendency toward cell-cycle delay. The reference drug displayed a significant trend toward S-phase arrest. Nonetheless, this pattern aligns with its known mechanism of action [60] and confirms that the experimental conditions were sufficient to detect cycle-related shifts. On the other hand, the negative control consisted of untreated cells, establishing the baseline nuclear morphology and DNA content. The vehicle control (DMSO) was directly compared with this group to verify that any alterations observed with the active compounds were not attributable to the solvent. As expected, DMSO-treated cells preserved both their cellular and nuclear integrity and maintained a normal cell-cycle distribution, confirming that the solvent did not contribute to the changes detected in the experimental treatments. Overall, the findings show that compound **6d** strongly affects nuclear morphology and DNA content, suggesting the activation of cell death–related mechanisms, whereas compounds **6e** produce only minimal and non-significant alterations.

#### 2.3.4. Wound healing assay.

**Fig 8:**
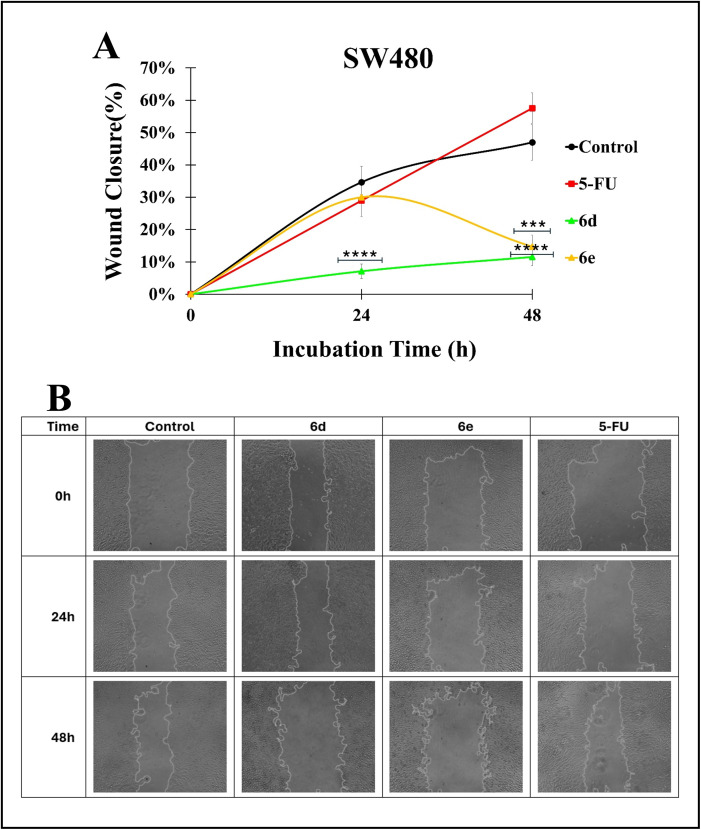
Effect of hybrids 6d and 6e on cell migration in SW480 cells. **A)** Percentage of wound closure at different time points. **B)** Cell scratch assay images. Magnification: 10X. Statistical analysis was performed by one-way ANOVA with Tukey’s test using Graphpad Prism 8.0.1 (***p < 0.001 and **** *p* < 0.0001), comparing against the control group.

Cell migration plays a crucial role in various physiological processes, including wound healing, angiogenesis, and tumor metastasis [61]. For this purpose, the wound-healing assay is a widely accepted *in vitro* method for assessing these key processes [62]. In this study, treatment with hybrids **6d** and **6e** resulted in a significant increase in wound width over time, in contrast to both the vehicle control and the reference drug 5-FU, suggesting that the test compounds may actively interfere with mechanisms underlying wound healing. This property could be related to different mechanisms that impair cytoskeletal organization, cell adhesion, or survival, which have been associated with several anticancer strategies, particularly in limiting metastatic potential. However, expanded wound areas may reflect not only impaired migration but also treatment-induced cytotoxicity, particularly when associated with apoptosis-related features such as membrane blebbing and nuclear condensation, as evidenced by the previous results (Fig 8). In contrast to several reports in the literature, our wound healing assay revealed that 5-fluorouracil (5-FU), at the tested concentration and exposure time, did not significantly inhibit the migration of colorectal cancer cells. This result diverges from previous findings, which have consistently demonstrated 5-FU’s antimigratory properties in colorectal models. For instance, some authors have reported that 5-FU effectively reduced cell migration in colorectal cancer cells, attributing this effect to its modulation of EMT-related pathways [63,64]. Similarly, Wang et al. [65] suggested that 5-FU exerts its antimigratory effects by suppressing epithelial–mesenchymal transition (EMT), and Seo et al. [66] linked its activity to the upregulation of antioxidant enzyme sestrin 2. However, variability in response may depend on multiple factors, including cell-line sensitivity, experimental conditions, treatment duration, and dosing regimen. Notably, our findings align with the notion that 5-FU’s primary mechanism of action is cytostatic and cytotoxic, rather than specifically targeting migratory pathways. Furthermore, the lack of inhibition observed in our study may reflect a concentration threshold below which the cells’ migratory machinery remains unaffected.

#### 2.3.5. Molecular Docking studies.

Fluorescence microscopy and flow cytometry analyses could indicate that the active hybrids **6d** and **6e** exert significant effects on cell integrity and cell-cycle progression. These findings are particularly interesting and are consistent with abundant previous reports when benzofuran and pterostilbene-based molecules have been widely reported to induce accumulation in the sub-G0/G1 phase through direct inhibition of CDK2, CDK4, and CDK6, as well as by modulating the intrinsic apoptotic pathway by influencing key pro-apoptotic regulators, including caspases, p53, PARP-1, MDM2, and TOP2A [11,36,67–73]. Guided by these observations, we conducted a multi-target molecular docking study to suggest a plausible molecular-level mechanism for the **6d** and **6e** hybrids. Specifically, we examined key interactions of the hybrids with the three-dimensional structures of proteins associated with the sub-G0/G1 phase (CDK2, CDK4, and CDK6), as well as with proteins involved in pro-apoptotic pathways, including caspases 3/7/8/9, mutant p53, PARP-1, MDM2, and TOP2A.

To conduct docking experiments, we first performed self-docking simulations to validate our AutoDock Vina docking protocol. To that, we carried out a comparison of the binding modes of the re-dock crystallographic drugs and their crystallographic binding modes deposited in each PDB archive. The results indicated that our docking procedure reproduced the binding modes of the co-crystallized inhibitors, with root-mean-square deviation (RMSD) values ranging from 0.9 to 1.083 Å. This finding indicates a high level of feasibility in our protein–ligand docking protocol. After the docking protocol was validated, compounds **6d** and **6e** hybrids was therefore docked into the each catalytic domain of X-ray crystallographic structures of CDK2 (PDB ID: 4kd1), CDK4 (PDB ID: 7sj3), and CDK6 (PDB ID: 5l2s), caspase-3 (PDB code: 5i9b), caspase-7 (PDB ID: 1f1j), caspase-8 (PDB ID: 3kjn), caspase-9 (PDB ID: 2ar9), PARP-1 (PDB ID: 4und), MDM2 (PDB ID: 4hg7), and TOP2A (PDB ID: 5gwk), and p53 (PDB ID: 1TSR) proteins via grid-based ligand docking with AutoDock Vina and affinity scores along with the best binding pose and protein–ligand interactions were examined (Table 3).

**Table 3:** The top-docking score (binding affinity, kcal/mol) of the hybrids 6d and 6e against multiple molecular targets.

Target protein	Ligands (docking score, kcal/mol)										
	6d	6e	Ac-DEVD-CMK^a^	MMX-9^b^	NSC194598^c^	SCH529074^d^	Talazoparib	Nutlin	Dinaciclib	Abemaciclib	Etoposide
Caspase−3	−8.1	−8.2	−8.2	--	--	--	--	--	--	--	
Caspase−7	−7.9	−7.9	−8.2	--	--	--	--	--	--	--	--
Caspase−8	−8.1	−8.1	--	−9.0	--	--	--	--	--	--	--
Caspase−9	−7.4	−7.8	--	−9.0	--	--	--	--	--	--	--
Mutant p53	**−10.7**	**−10.8**	--	--	−7.3	−7.0	--	--	--	--	--
PARP-1	**−10.5**	**−10.4**	--	--	--	--	−11.0	--	--	--	--
MDM2	−8.5	−8.4	--	--	--	--	--	−7.9	--	--	--
CDK2	−9.4	−9.3	--	--	--	--	--	--	−9.6	--	--
CDK4	**−10.1**	**−10.2**	--	--	--	--	--	--	--	−11.1	--
CDK6	−9.1	−9.2	--	--	--	--	--	--	--	−11.0	--
TOP2A	−7.9	−7.8	--	--	--	--	--	--	--	--	−9.1

Bold values indicate the best binding energy scores. Poly(ADP-ribose) Polymerase (PARP). ^a^Potent cell-permeable and irreversible inhibitor of caspase-3/7; ^b^Irreversible peptidomimetic inhibitor of caspase-8. ^c^Quinazolin-based p53-DNA-binding inhibitor; ^d^Potent and orally p53-DNA noncompetitive activator of mutant p53, binding to p53 DNA binding domain

**Fig 9:**
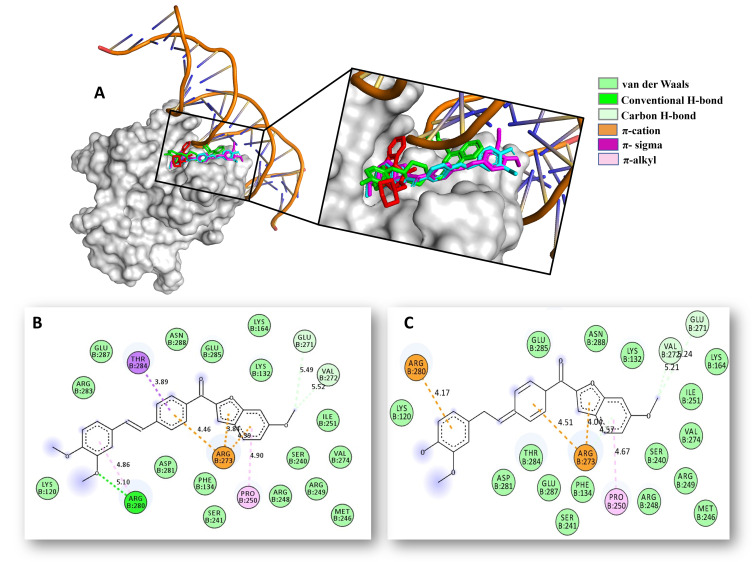
Docking studies of 6d/6e-p53 complex. **A)** 3D superposition of the best-docked pose of p53-.

**Fig 10:**
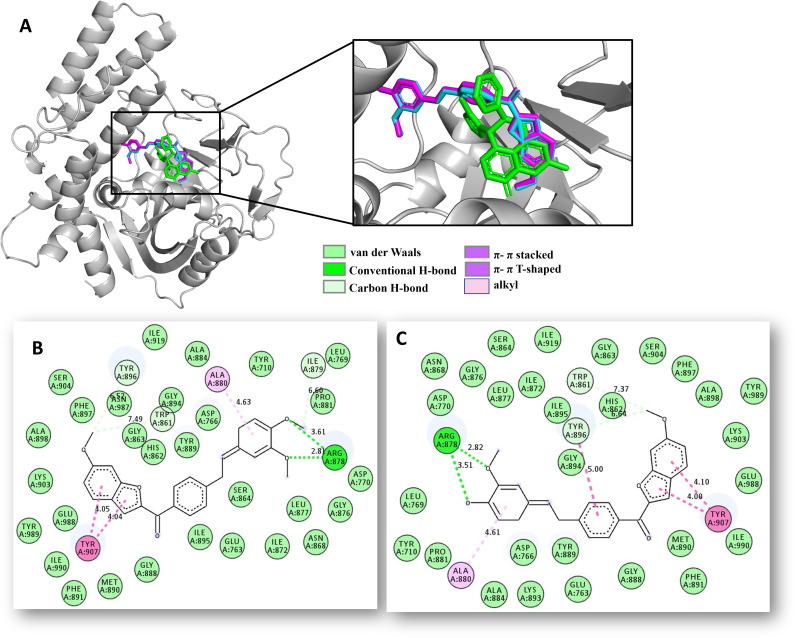
Docking studies of 6d/6e-PARP-1 complex. **A)** 3D superposition of the best-docked pose of PARP-1/Talazoparib (in green), **6d** (in magenta), **6e** (in cyan) into the Talazoparib binding site of PARP-1. **B)** 2D interaction binding plot between **6d** and PARP-1 protein. **C)** 2D interaction binding plot between **6e** and PARP-1 protein.

**Fig 11:**
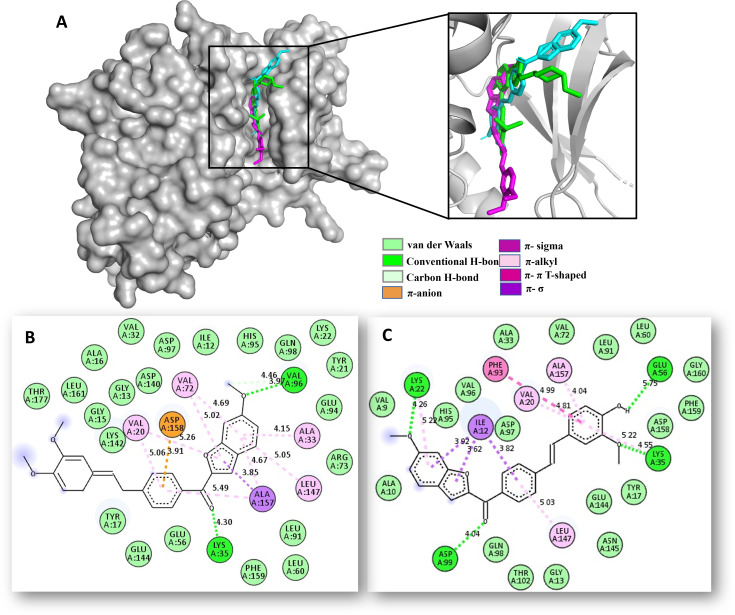
Docking studies of 6d/6e-CDK4 complex. **A)** 3D superposition of the best-docked pose of CDK4/Abemaciclib (in green), **6d** (in magenta), **6e** (in cyan) into the Abemaciclib binding site of CDK4. **B)** 2D interaction binding plot between **6d** and CDK4 protein. **C)** 2D interaction binding plot between **6e** and CDK4 protein.

According to the results, greater binding affinity values were obtained after docking action in hybrids **6d** and **6e** with mutant p53, with a docking score around −10.8 kcal/mol, followed by PARP-1 (−10.5 kcal/mol), and CDK4 (−10.2 kcal/mol), which were notably higher or comparable to crystallographic drugs. These computational findings support our experimental evidence suggesting that hybrids **6d** and **6e** could prevent cell proliferation in colorectal cancer cells by activating cell death–related mechanisms associated with arrested cells in the G0/G1 phase and by inducing apoptosis via the mitochondria-mediated intrinsic pathway. In addition to binding scores, our modeling work also suggested that, similar to co-crystallized inhibitors, **6d** and **6e** bind to mutant p53, CDK4, and PARP-1, respectively, through several non-covalent interactions with those essential amino acid residues required for their functional roles (Fig 9-11).

Thus, 1) For mutant p53 protein, hybrids **6d** and **6e** were positioning between the H2 helix and L3 loop of p53 protein harboring the DNA-binding domain of p53 (Lys120, Ser241, Arg248, Arg273, Ala276, Cys277, Arg280, Asp281, Arg283, Thr284). The results showed that compounds **6d** and **6e** are not only capable of binding to p53 with significantly better binding affinity (~−10.8 kcal/mol) than those current inhibitors SCH529074 (−7.0 kcal/mol; in green) and NSC194598 (−7.3 kcal/mol, in red), but are also well-accommodated into the p53/DNA-binding interface (Fig 9A), establishing numerous contacts (hydrogen bonds, π-cation, π-σ, π-alkyl, van der Waals) with the critical residues Lys120, Arg248, Arg273, Arg280, Arg283, and Thr284 (Fig 9B, C). According to experimental and docking evidence, modulation of p53 function may be a mechanism by which hybrids **6d** and **6e** triggered cell death in CRC cells (Fig 9).

2) For the PARP-1 protein, hybrids **6d** and **6e** were positioned into the Talazoparib binding site, which is constituted by eight hot spot amino acid residues (Glu763, Asp766, Gly863, Arg878, Tyr896, Ser904, Tyr907, and Glu988), which are critical for the biological function of PARP-1. Molecular docking results

suggested that **6d** and **6e** would bind to PARP-1 with similar affinity to the drug Talazoparib (−11.0 kcal/mol) in about −10.5 kcal/mol, fitting well within the Talazoparib-site (Fig 10A), establishing critical contacts (hydrogen bonds, π-π stacked, π-π T-shaped, alkyl, van der Waals) with two crucial residues as Arg878 and Tyr907 (Fig 10B, 10C). These findings could apparently explain how hybrids **6d** and **6e** can stop cell growth and proliferation in SW480 cancer cells

3) For the CDK4 protein, hybrids **6d** and **6e** were positioned into the ATP-binding pocket, which is constituted by thirteen hot spot amino acid residues (Ile12, Tyr17, Val20, Ala33, Lys35, Glu56, Phe93, His95, Val96, Leu147, Ala157, and Asp158), which play a crucial role in the function of PARP-1 protein. The results indicate that compounds **6d** and **6e** not only exhibit binding affinities to CDK4 (~−10.2 kcal/mol) comparable to that of the reference drug Abemaciclib (−11.1 kcal/mol; shown in green), but also fit well within the CDK4/abemaciclib binding pocket (Fig 11A). Within this pocket, they establish multiple stabilizing interactions including hydrogen bonds, π- π T-shaped, π–anion, π–σ, π–alkyl, and van der Waals contacts with most of key residues such as Ile12, Val20, Ala33, Lys35, Glu56, Phe93, Val96, Leu147, Ala157, and Asp158 (Fig 11B, C). These computational findings also suggest that direct inhibition of CDK4 function may be a plausible mechanism by which **6d** and **6e** induce cell death in CRC cells (Fig 11).

Overall, the computational results were in good agreement with the *in vitro* biological data, allowing us to infer that the cytotoxic and antiproliferative effects of the evaluated hybrids in the human colon cancer cell line SW480 may be associated with their ability to interact with multiple colorectal cancer–relevant targets, particularly mutant p53, CDK4, and PARP-1.

modulators SCH529074 (in green), NSC194598 (in red), **6d** (in magenta), **6e** (in cyan) into the Talazoparib binding site of p53. B) 2D interaction binding plot between **6d** and p53 protein. C) 2D interaction binding plot between **6e** and p53 protein.

### 2.4. ADME/*Tox* modelling studies

#### 2.4.1. Drug-likeness/pharmacokinetic studies.

In the search for new drug candidates, the evaluation of physicochemical and pharmacokinetic parameters is essential, as these factors critically influence solubility, ionization, and biological activity. In modern drug discovery, a variety of rule-based filters have been developed to define drug-like prototypes using fundamental molecular descriptors derived from chemical structures. This is particularly relevant to the development of novel anticancer agents, in which key pharmacokinetic indices have gained prominence over the past decade. Computational tools have emerged as efficient, cost-effective, and rapid means to assess these properties. In this context, the most promising hybrids, compounds **6d** and **6e**, were subjected to *in silico* analysis to evaluate their biopharmaceutical profiles and drug-likeness. Specifically, 13 drug-like parameters associated with oral bioavailability were calculated using SwissADME, and the results are summarized in Table 4.

**Table 4:** Biopharmaceutical and drug-likeness profiles for hit hybrids 6d and 6e.

Property	6d	6e
MW ^a^	414.457	400.430
TPSA ^b^	57.90	68.90
n-RotBond	7	6
n-ON ^c^	4	5
n-OHNH ^d^	0	1
log P_o/w_^e^	4.96	4.71
logK_HSA_^f^	0.750	0.732
Fsp^3 g^	0.12	0.08
#ArRNG ^h^	3	3
Caco-2 (nm/s) ^i^	3409	1098
App. MDCK (nm/s) ^j^	1862	547
%GI^k^	>80%	>80%
BBB permeant^l^	No/ Inactive^m^	No/ Inactive^m^

^a^Molecular weight distribution of traded drugs peaked in the 200−600 g/mol range. ^b^ Polar surface area (*PSA*, Å^2^) (<140 Å^2^). ^c^ *n-ON* number of hydrogen bond acceptors <10. ^d^ n-*OHNH* number of hydrogen bond donors ≤5. ^e^ Octanol-water partition coefficient (*log P*_*o/w*_) (–2.0 to +6.5). ^f^ Binding-serum albumin (*LogK*_*HSA*_) (−1.5 to +1.2). ^g^ Fraction of sp^3^ carbon atoms (Optimal: Fsp^3^ < 0.5). ^h^ The number of aromatic/heteroaromatic rings (Optimal: ≤ 3). ^i^ Human intestinal permeation (<25 poor, > 500 great). ^j^ Madin-Darby canine kidney (*MDCK*) cells permeation (<25 poor, > 500 great). ^k^ % Human oral gastrointestinal (*GI*) absorption (<25% is poor, > 80% is high). ^l^ The probability of a good BBB crossing blood-brain barrier (BBB) permeation both consist in the readout of the BOILED-Egg model ^m^ calculated by ProTox 3.0 (https://tox.charite.de/protox3/).

The *in-silico* analysis would suggest that hybrids **6d** and **6e** exhibit favorable drug-like profiles comparable to major orally traded drugs. Their predicted lipophilicity values (as logP_o/w_) of approximately 4.96 and 4.71, respectively, fall within the optimal range for lipid-based formulations (–2.0 to +6.0) [74]. Furthermore, both compounds demonstrated high predicted human gastrointestinal absorption (%GI > 80%), suggesting efficient uptake following oral administration. Supporting this, the apparent permeability values through Caco-2/MDCK cell monolayers, commonly used models for intestinal absorption, were estimated at 3409/1862 nm/s for **6d** and 1098/547 nm/s for **6e**, indicating good permeability potential [75–77].

Additionally, both compounds presented an ideal topological polar surface area (TPSA) of 57.90 and 68.90 Å², respectively, which apparently will favor membrane permeability and oral bioavailability [78]. Human serum albumin binding (as logK_HSA_) values were calculated at 0.750 for **6d** and 0.732 for **6e**, aligning well with the typical therapeutic range (–1.5 to +1.5) [79,80], suggesting suitable systemic distribution. Structural features such as the fraction of sp³-hybridized carbons (Fsp^3^ = 0.12 and 0.08, respectively) and the presence of three aromatic/heteroaromatic rings also fit established drug-likeness criteria [81–84]. Finally, the blood–brain barrier (BBB) permeability predictions indicated limited brain penetration for both compounds, reducing the chance of central nervous system side effects. Collectively, these findings highlight **6d** and **6e** as promising candidates for further preclinical development.

#### 2.4.2. Bioavailability radar model for compounds 6d and 6e.

**Fig 12:**
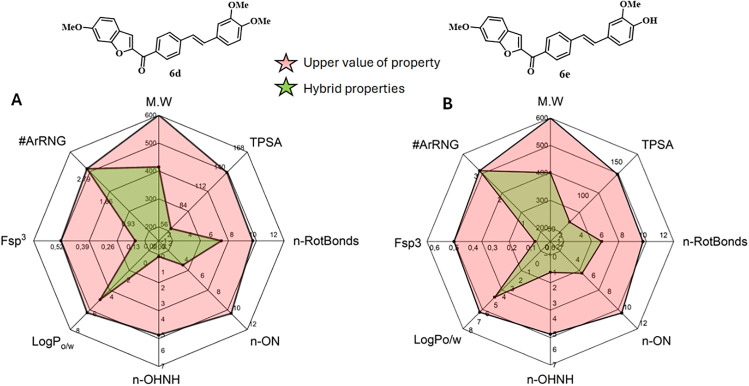
Bioavailability radar plot for promising 6d and 6e.

The bioavailability radar plot offers a visual representation of key molecular descriptors relevant to oral drug-likeness, including size, polarity, solubility, saturation, flexibility, and lipophilicity [85]. In this study, eight critical parameters were evaluated for compounds **6d** and **6e** using radar mapping. As shown in **Fig 12**, the computed properties for both hybrids (highlighted in green) fall within the optimal drug-like space (indicated in pink). The close alignment of these values with the recommended ranges suggests that both compounds exhibit favorable oral bioavailability profiles and may be strong candidates for progression into advanced preclinical evaluation.

#### 2.4.3. *In silico* toxicological evaluation and safety assessment.

In addition to pharmacokinetic profiling, a comprehensive *in silico* toxicity assessment was conducted for lead compounds **6d** and **6e**. Multiple open-access computational tools, including ToxTree, Pred-hERG, ProTox 3.0, TEST, OSIRIS, pkCSM, ADMET-SAR, and SwissADME, were employed to predict potential toxicological liabilities. Early toxicological evaluation plays a critical role in preclinical drug development, and chemoinformatics approaches provide a rapid, cost-effective alternative to experimental testing [86,87]. The *in silico* analyses revealed no indications of tumorigenic, mutagenic, immunotoxic, nephrotoxic, neurotoxic, hepatotoxic, cardiotoxic, or irritant effects for either compound. Furthermore, no structural alerts were detected for reproductive toxicity, covalent DNA binding, or oral toxicity. Importantly, both hybrids also tested negative for pan-assay interference compounds (PAINS), suggesting a low risk of promiscuity.

Taken together, this study presents, for the first time, novel anticancer candidates combining the pterostilbene scaffold and the benzofuran moiety within a single molecular framework, exhibiting apparently ideal pharmacokinetic and toxicological profiles. In light of these findings, lead compounds **6d** and **6e** emerge as promising candidates for further investigation in the context of colorectal cancer therapy.

## 3. Conclusion

This study provides valuable insight into the biological activity of a novel series of benzofuran-pterostilbene conjugates against colorectal adenocarcinoma cells. The compounds demonstrated significant antiproliferative effects, with cytotoxicity evidenced by morphological alterations, including chromatin condensation, nuclear fragmentation, and disrupted mitochondrial membrane potential, as indicated by Hoechst, DiOC6, and propidium iodide staining. Furthermore, the wound-healing assay revealed a notable inhibition of cell migration in the groups treated with hybrids **6d** and **6e**, which also led to a widening of the wound gap, suggesting impaired cell motility and/or compromised viability. The docking studies were in good agreement with the experimental assays, suggesting that the in vitro cytotoxic effects observed for compounds **6d** and **6e** in SW480 colon carcinoma cells may be strongly associated with a multitarget mechanism involving mutant p53, CDK4, and PARP-1. Nevertheless, further complementary studies are required to elucidate the precise mechanisms of action of these hybrid molecules. Finally, ADME-tox modelling studies suggest that these two hybrids have favorable pharmacokinetic indices, optimal drug-like properties, and a safer toxicological profile. Collectively, these results underscore the potential of the tested compounds as promising candidates for the design of new therapeutic alternatives for colorectal cancer, highlighting the need for further studies to elucidate their mechanisms of action, evaluate their in vivo efficacy, and establish their therapeutic potential and clinical relevance.

## 4. Experimental section

### 4.4. Chemical synthesis

#### 4.4.2. General information.

Microwave reactions were conducted using a CEM Discover microwave reactor with sealed vessels (monowave, maximum power 300 W, temperature control by IR sensor, and fixed temperature). The Varian equipment was used to record the ^1^H and ^13^C NMR spectra at 300 and 75 MHz, respectively. The DMSO-*d*_6_ or CDCl_3_ signals were utilized as references. The solvent peak serves as a reference, and TMS as an internal standard for chemical shifts (δ), which are represented in parts per million (ppm); coupling constants (J) are provided in Hertz (Hz). The concentration used for each sample was between 0.03 and 0.05 mg/uL. A Bruker Impact II UHR-Q-TOF mass spectrometer (Bruker Daltonik GmbH, Bremen, Germany) operating in positive mode was used to obtain HRMS.

#### 4.4.3. Synthesis of intermediates 2 and 4.

*Synthesis of 2-bromo-1-(4-bromophenyl)ethan-1-one (2).* In a 10 mL flask with a flat bottom and a magnetic stirring bar, 4-bromoacetophenone (1 mmol), N-bromosuccinimide (1.2 mmol), p-toluenesulfonic acid (0.1 mmol), and CHCl3 (5 mL) were added. After stirring and being exposed to microwave radiation for two hours, the mixture was heated to 70°C at 100W. After the reaction mixture cooled and the solvent evaporated, the crude residue was purified by column chromatography on silica gel, eluting with mixtures of hexane and ethyl acetate in varying ratios. 90% yield.

90% yield. White solid, m.p. 98–102°C; ^**1**^**H-NMR (300 MHz, CDCl**_**3**_) δ 7.90 (d, *J* = 8.6 Hz, 2H, (2 and 6)), 7.69 (d, *J* = 8.6 Hz, 2H, (3 and 5)), 4.45 (s, 2H (2)). ^**13**^**C-NMR (75 MHz, CDCl**_**3**_) δ 190.47 (C = O), 132.63 (1’), 132.27 (3 and 5), 130.47 (2 and 6), 129.37 (4), 30.46 (-CH_2_-Br).

*Synthesis of* (4-bromophenyl)(6-methoxybenzofuran-2-yl)methanone (**4**): In a 35 mL flat-bottomed flask equipped with a magnetic stirring bar, were placed 1eq of 2-hydroxy-4-methoxybenzaldehyde, 1.5 eq of 2-bromo-1-(4-bromophenyl)ethan-1-one (**2**), 3 eq of K_2_CO_3_, and 5 mL of dry DMF. This mixture was irradiated for 5 min at 300 W and 180 °C in the CEM reactor. Then, the reaction product was placed over water ice. The solid obtained was filtered under reduced pressure, washed several times with cold water, and dried.

Light yellow solid; Yield: 90%; M.p. 200–202 °C; ^**1**^**H NMR (600 MHz, CDCl**_**3**_) δ 7.92 (d, *J* = 8.6 Hz, 2H, (2 and 6)), 7.68 (d, *J* = 8.5 Hz, 2H, (3 and 5)), 7.60 (d, *J* = 8.7 Hz, 1H, (4-benzofuran)), 7.49 (s, 1H, (3-benzofuran)), 7.08 (d, *J* = 2.2 Hz, 1H, (7-benzofuran)), 6.99 (dd, *J* = 8.7, 2.2 Hz, 1H, (5-benzofuran)). ^**13**^**C NMR (75 MHz, CDCl**_**3**_) δ 182.66 (C = O), 161.46 (6-benzofuran), 157.77 (7a-benzofuran), 151.65 (2-benzofuran), 136.21 (1), 131.85 (3 and 5)), 130.91 (2 and 6), 127.74 (4), 123.76 (3a-benzofuran), 120.30 (4-benzofuran), 117.40 (2-benzofuran), 114.79 (5-benzofuran), 95.61 (7-benzofuran), 55.81 (OMe).

*Styrene synthesis (****5a-j****):* The styrenes were produced using the previously mentioned Knoevenagel-Doebner condensation processes or the Wittig Reaction in a microwave [42–44].

#### 4.4.4. Synthesis of hybrids 6a-h.

*General procedure for the Mizoroki-Heck reactions (compounds* ***6a-h****):* Compound 3 (0.3 mmol, 100 mg), pyrrolidine (0.6 mmol, 50 µL), Pd(OAc)2 (6 mg, 0.027 mmol), styrene (0.3 mmol) and DMF (1 mL) were added to a 10 mL flask with a flat bottom and a magnetic stirring bar. The mixture was then heated at 165°C and 200W for 2 h under microwave irradiation. After the mixture was allowed to cool, 100 mL of 5% HCl was added. After being filtered under low pressure, the resulting solid was washed three times with cold distilled water. Ultimately, column chromatography (silica gel, hexane-ethyl acetate combination) was used to purify the solid, yielding the coupling products in a 30–80% yield.

*(E)-(4-(2,4-dimetoxiestiril)fenil)(6-metoxibenzofuran-2-il)metanona (* ***6a*** *):*

Solid light yellow; Yield: 34%; M.p. 118–121 °C. ^**1**^**H NMR (300 MHz, CDCl**_**3**_) δ 8.02 (d, *J* = 8.4 Hz, 2H, (2 and 6-ring A)), 7.63 (d, *J* = 8.3 Hz, 2H, (3 and 5-ring A)), 7.58 (d, *J* = 8.7 Hz, 1H, (4-benzofuran)), 7.55 (d, *J* = 16.5 Hz, 1H, (*E*-styryl)), 7.54 (d, *J* = 8.2 Hz, 1H, (6-ring B)), 7.48 (d, *J* = 0.9 Hz, 1H, (3-benzofuran), 7.11 (d, *J* = 2.3 Hz, 1H, (7-benzofuran), 7.07 (d, *J* = 16.5 Hz, 1H, (*E*-styryl)), 6.96 (dd, *J* = 8.6, 2.2 Hz, 1H, (5-benzofuran)), 6.54 (dd, *J* = 8.5, 2.4 Hz, 1H, (5-ring B)), 6.49 (d, *J* = 2.4 Hz, 1H, (3-ring B)), 3.89 (2 x OMe), 3.84 (s, OMe). ^**13**^**C NMR (75 MHz, CDCl**_**3**_) δ 183.26 (C = O), 161.22 (4-ring B), 158.50 (2-ring B), 157.65 (6-benzofuran), 152.23 (7a-benzofuran), 143.03 (2-benzofuran), 135.63 (1-ring A), 132.03 (4-ring A), 130.04 (2 and 6-ring A), 127.76 (Ar_1_-**C**H = CH-Ar_2_), 126.31 (6-ring B), 126.24 (3 and 5-ring A), 125.72 (Ar_1_-CH = **C**H-Ar_2_), 123.69 (3a-benzofuran), 120.53 (4-benzofuran), 118.99 (1-ring B), 116.89 (3-benzofuran), 114.54 (5-benzofuran), 105.25 (5-ring B), 98.57 (3-ring B), 95.74 (7-benzofuran), 55.85 (OMe), 55.65 (OMe), 55.55 (OMe). ESI-MS (*m/z*): 415,1540 [M + H]^+^ calcd for C_26_H_22_O_5_ [M + H]^+^ 415,1560.

*E)-(4-(2,5-dimethoxystyryl)phenyl)(6-methoxybenzofuran-2-yl)methanone (****6b***)

Solid light yellow; Yield: 37%; M.p. 115–118 °C. ^**1**^**H NMR (300 MHz, CDCl**_**3**_) δ 8.03 (d, *J* = 8.3 Hz, 2H (2 and 6-ring A)), 7.66 (d, *J* = 8.3 Hz, 2H (3 and 5-ring A)), 7.61 (d, *J* = 16.4 Hz, 1H, (*E*-styryl)), 7.58 (d, *J* = 8.6 Hz, 1H (4-benzofuran)), 7.48 (s_app_, 1H (3-benzofuran)), 7.18 (d, *J* = 2.8 Hz, 1H (6-ring B)), 7.16 (d, *J* = 16.4 Hz, 1H, (*E*-styryl)), 7.11 (d, *J* = 2.6 Hz, 1H (7-benzofuran)), 6.97 (dd, *J* = 8.7, 2.2 Hz, 1H (5-benzofuran)), 6.86–6.84 (m, 2H (3 and 4-ring B), 3.89 (s, OMe), 3.87 (s, OMe), 3.83 (s, OMe). ^**13**^**C NMR (75 MHz, CDCl**_**3**_) δ 183.19 (C = O), 161.20 (6-benzofuran), 157.62 (7a-benzofuran), 153.77 (5-ring B), 152.10 (2-benzofuran), 151.76 (2-ring B), 142.29 (4-ring A), 136.09 (1-ring A), 129.96 (2 and 6-ring A), 128.09 (Ar_1_-**C**H = CH-Ar_2_), 126.56 (1-ring B), 126.54 (3 and 5-ring A), 126.18 (Ar_1_-CH = **C**H-Ar_2_), 123.66 (3a-benzofuran), 120.44 (4-benzofuran), 116.95 (4-ring B), 114.57 (3-benzofuran), 114.52 (6-ring B), 112.33 (3-ring B), 111.85 (5-benzofuran), 95.67 (7-benzofuran), 56.27 (OMe), 55.81 (2 x OMe). ESI-MS (*m/z*): 415,1540 [M + H]^+^ calcd for C_26_H_22_O_5_ [M + H]^+^ 415,1562.

*(E)-(4-(3,5-dimethoxystyryl)phenyl)(6-methoxybenzofuran-2-yl)methanone (* ***6c*** *):*

Solid yellow; Yield: 24%; M.p. 128–129 °C. ^**1**^**H NMR (300 MHz, CDCl**_**3**_) δ 8.04 (d, *J* = 8.2 Hz, 2H (2 and 6-ring A)), 7.64 (d, *J* = 8.6 Hz, 2H (3 and 5-ring A)), 7.58 (d, *J* = 8.7 Hz, 1H (4-benzofuran)), 7.49 (s_app_, 1H (3-benzofuran)), 7.19 (d, *J* = 16.3 Hz, 1H (*E*-styryl)), 7.14 (d, *J* = 16.4 Hz, 1H, (*E*-styryl)), 7.11 (s_app_, 1H (7-benzofuran)), 6.97 (dd, *J* = 8.7, 2.2 Hz, 1H (5-benzofuran)), 6.71 (d, *J* = 2.3 Hz, 2H (2 and 6-ring B)), 6.44 (t, *J* = 2.2 Hz, 1H (4-ring B)), 3.89 (s, OMe), 3.85 (s, 2 x OMe). ^**13**^**C NMR (75 MHz, CDCl**_**3**_) δ 183.23 (C = O),161.33 (6-benzofuran), 161.17 (3 and 5- ring B), 157.74 (7a-benzofuran), 152.12 (2-benzofuran), 141.63 (4-ring A), 138.83 (1-ring B), 136.46 (1-ring A), 131.53 (Ar_1_-**C**H = CH-Ar_2_), 130.09 (2 and 6-ring A), 128.12 (Ar_1_-CH = **C**H-Ar_2_), 126.62 (3 and 5-ring A), 123.76 (3a-benzofuran), 120.51 (4-benzofuran), 117.12 (3-benzofuran), 114.66 (5-benzofuran), 105.02 (2 and 6-ring B), 100.71 (4-ring B), 95.75 (7-benzofuran), 55.89 (OMe), 55.55 (2 x OMe). ESI-MS (*m/z*): 415,1540 [M + H]^+^ calcd for C_26_H_22_O_5_ [M + H]^+^ 415,1561.

*(E)-(4-(3,4-dimetoxiestiril)fenil)(6-metoxibenzofuran-2-il)metanona (* ***6d*** *):*

Solid light yellow; Yield: 65%; M.p. 150−154°C. ^**1**^**H NMR (300 MHz, CDCl**_**3**_) δ 8.04 (d, *J* = 8.3 Hz, 2H, (2 and 6-ring A)), 7.63 (d, *J* = 8.3 Hz, 2H, (3 and 5-ring A)), 7.59 (d, *J* = 8.6 Hz, 1H, (4-benzofuran)), 7.50 (s_app_, 1H, (3-benzofuran)), 7.21 (d, *J* = 16.2 Hz, 1H, (*E*-styryl)), 7.12 (d, *J* = 2.3 Hz, 2H, (7-benzofuran)), 7.12–7.09 (m, 2H, (2 and 6-ring B)), 7.04 (d, *J* = 16.2 Hz, 1H, (*E*-styryl)), 6.97 (dd, *J* = 8.7, 2.2 Hz, 1H, (5-benzofuran)), 6.89 (d, *J* = 8.7 Hz, 1H, (5-ring B)), 3.97 (s, OMe), 3.92 (s, OMe), 3.90 (s, OMe). ^**13**^**C NMR (75 MHz, CDCl**_**3**_) δ 183.27 (C = O), 161.32 (6-benzofuran), 157.73 (7a-benzofuran), 152.19 (2-benzofuran), 149.66 (3-ring B), 149.34 (4-ring B), 142.16 (4-ring A), 136.04 (1-ring A), 131.42 (1-ring B), 130.15 (2 and 6-ring A), 129.96 (Ar_1_-**C**H = CH-Ar_2_), 126.30 (3 and 5-ring A), 125.68 (Ar_1_-CH = **C**H-Ar_2_), 123.75 (3a-benzofuran), 120.68 (4-benzofuran), 120.54 (6-ring B), 117.05 (3-benzofuran), 114.65 (5-benzofuran), 111.34 (5-ring B), 109.00 (2-ring B), 95.78 (7-benzofuran), 56.11 (OMe), 56.06 (OMe), 55.91 (OMe). ESI-MS (*m/z*): 415,1540 [M + H]^+^ calcd for C_26_H_22_O_5_ [M + H]^+^ 415,1559.

*(E)-(4-(4-hidroxi-3-metoxiestiril)fenil)(6-metoxibenzofuran-2-il)metanona (* ***6e*** *):*

Solid orange; Yield: 20%; M.p. 133–136°C. ^1^H NMR (300 MHz, **CDCl**_**3**_) δ 8.03 (d, *J* = 8.3 Hz, 2H, (2 and 6-ring A)), 7.62 (d, *J* = 8.3 Hz, 2H, (3 and 5-ring A)), 7.58 (d, *J* = 8.6 Hz, 1H, (4-benzofuran)), 7.49 (d, *J* = 1.0 Hz, 1H, (3-benzofuran)), 7.19 (d, *J* = 16.4 Hz, 1H, (*E*-styryl)), 7.11 (d, *J* = 3.2 Hz, 1H, (7-benzofuran)), 7.09–7.06 (m, 2H, (2 and 6-ring B)), 7.01 (d, *J* = 16.4 Hz, 1H, (*E*-styryl)), 6.97 (dd, *J* = 8.7, 2.2 Hz, 1H, (5-benzofuran)), 6.94 (d, *J* = 8.6 Hz, 1H, (5-ring B)), 3.97 (s, OMe), 3.90 (s, OMe). ^13^C NMR **(75 MHz, CDCl**_**3**_) δ 183.12 (C = O), 161.19 (6-benzofuran), 157.61 (7a-benzofuran), 152.08 (2-benzofuran), 146.84 (4-ring B), 146.31 (3-ring B), 142.09 (4-ring A), 135.87 (1-ring A), 131.47 (Ar_1_-**C**H = CH-Ar_2_), 130.03 (2 and 6-ring A), 129.38 (Ar_1_-CH = **C**H-Ar_2_), 126.14 (3 and 5-ring A), 125.24 (3a-benzofuran), 123.63 (1-ring B), 121.11 (4-benzofuran), 120.43 (6-ring B), 116.91 (5-ring B), 114.74 (3-benzofuran), 114.53 (5-benzofuran), 108.46 (2-ring B), 95.67 (7-benzofuran), 56.00 (OMe), 55.80 (OMe). ESI-MS (*m/z*): 401,1383 [M + H]^+^ calcd for C_25_H_20_O_5_ [M + H]^+^ 401,1393.

*(E)-(6-metoxibenzofuran-2-il)(4-(2,4,5-trimetoxiestiril)fenil)metanona (* ***6f*** *):*

Solid yellow; Yield: 34%; M.p. 131–132°C. ^**1**^**H NMR (300 MHz, CDCl**_**3**_) δ 8.03 (d, *J* = 8.3 Hz, 2H, (2 and 6-ring A)), 7.64 (d, *J* = 8.3 Hz, 2H, (3 and 5-ring A)), 7.59 (d, *J* = 16.4 Hz, 1H, (*E*-styryl)), 7.58 (d, *J* = 8.6 Hz, 1H, (4-benzofuran)), 7.48 (s, 1H, (3-benzofuran)), 7.15 (s, 1H, (6-ring B)), 7.11 (d, *J* = 2.2 Hz, 1H, (7-benzofuran)), 7.04 (d, *J* = 16.4 Hz, 1H, (*E*-styryl)), 6.97 (dd, *J* = 8.7, 2.2 Hz, 1H, (5-benzofuran)), 6.55 (s, 1H, (3-ring B)), 3.93 (OMe), 3.93 (OMe), 3.90 (OMe), 3.90 (OMe). ^**13**^**C NMR (75 MHz, CDCl**_**3**_) δ 182.52 (C = O), 161.15 (6-benzofuran), 157.58 (7a-benzofuran), 152.26 (2-benzofuran), 152.14 (4-ring B), 150.37 (2-ring B), 143.48 (5-ring B), 142.75 (4-ring A), 135.65 (1-ring A), 129.99 (2 and 6 ring A), 126.18 (3 and 5 ring A), 125.90 (Ar_1_-**C**H = CH-Ar_2_), 125.47 (Ar_1_-CH = **C**H-Ar_2_), 123.61 (4-benzofuran), 120.45 (3a-benzofuran), 117.54 (1-ring B), 116.81 (3-benzofuran), 114.48 (5-benzofuran), 109.47 (6-ring B), 97.42 (3-ring B), 95.68 (7-benzofuran), 56.65 (OMe), 56.61 (OMe), 56.13 (OMe), 55.79 (OMe). ESI-MS (*m/z*): 445,1646 [M + H]^+^ calcd for C_27_H_24_O_6_ [M + H]^+^ 445,1667.

*(E)-(6-metoxibenzofuran-2-il)(4-(3,4,5-trimetoxiestiril)fenil)metanona (* ***6g*** *):*

Solid light yellow; Yield: 32%; M.p. 145–147°C. ^**1**^**H NMR (300 MHz, CDCl**_**3**_) δ 8.04 (d, *J* = 8.3 Hz, 2H, (2 and 6-ring A)), 7.64 (d, *J* = 8.5 Hz, 2H, (3 and 5-ring A)), 7.59 (d, *J* = 8.6 Hz, 1H, (4-benzofuran)), 7.50 (s, 1H, (3-benzofuran)), 7.19 (d, *J* = 16.2 Hz, 1H, (*E*-styryl)), 7.11 (s, 1H, (7-benzofuran)), 7.07 (d, *J* = 16.2 Hz, 1H, (*E*-styryl)), 6.97 (dd, *J* = 8.7, 2.2 Hz, 1H, (5-benzofuran)), 6.79 (s, 2H, (2 and 6-ring B)), 3.94 (2 x OMe), 3.90 (OMe), 3.89 (OMe). ^**13**^**C NMR (75 MHz, CDCl**_**3**_) δ 183.10 (C = O), 161.23 (6-benzofuran), 157.64 (7a-benzofuran), 153.50 (3 and 5-ring B), 152.03 (2-benzofuran), 141.64 (4-ring A), 138.53 (4-ring B), 136.22 (1 ring A), 132.46 (1-ring B), 131.42 (Ar_1_-**C**H = CH-Ar_2_), 130.03 (2 and 6 ring A), 126.97 (Ar_1_-CH = **C**H-Ar_2_), 126.36 (3 and 5 ring A), 123.65 (4-benzofuran), 120.41 (3a-benzofuran), 116.99 (3-benzofuran), 114.56 (5-benzofuran), 103.94 (2 and 6-ring B), 95.67 (7-benzofuran), 61.04 (OMe), 56.21 (2 x OMe), 55.80 (OMe). ESI-MS (*m/z*): 445,1646 [M + H]^+^ calcd for C_27_H_24_O_6_ [M + H]^+^ 445,1663.

*(E)-(4-(4-hidroxi-3,5-dimetoxiestiril)fenil)(6-metoxibenzofuran-2-il)metanona (* ***6h*** *):*

Solid orange; Yield: 46%; M.p. 99–101°C. ^**1**^**H NMR (300 MHz, CDCl**_**3**_) δ 8.04 (d, *J* = 8.3 Hz, 2H, (2 and 6-ring A)), 7.63 (d, *J* = 8.3 Hz, 2H, (3 and 5-ring A)), 7.59 (d, *J* = 8.7 Hz, 1H, (4-benzofuran)), 7.50 (s, 1H, (3-benzofuran)), 7.18 (d, *J* = 16.2 Hz, 1H, (*E*-styryl)), 7.12 (s, 1H, (7-benzofuran)), 7.03 (d, *J* = 16.2 Hz, 1H, (*E*-styryl)), 6.98 (dd, *J* = 8.7, 2.3 Hz, 1H, (5-benzofuran)), 6.81 (s, 2H, (2 and 6-ring B)), 3.97 (s, 2 x OMe), 3.90 (s, OMe). ^**13**^**C NMR (75 MHz, CDCl**_**3**_) δ 183.10 (C = O), 161.21 (6-benzofuran), 157.62 (7a-benzofuran), 152.07 (2-benzofuran), 147.29 (3 and 5-ring B), 141.92 (4-ring A), 135.98 (4-ring B), 135.45 (1-ring A), 131.64 (Ar_1_-**C**H = CH-Ar_2_), 130.04 (2 and 6-ring A), 128.38 (1-ring B), 126.18 (3 and 5-ring A), 125.61 (Ar_1_-CH = **C**H-Ar_2_), 123.63 (4-benzofuran), 120.42 (3a-benzofuran), 116.91 (3-benzofuran), 114.54 (5-benzofuran), 103.73 (2 and 6-ring B), 95.68 (7-benzofuran), 56.39 (2 x OMe), 55.80 (OMe). ESI-MS (*m/z*): 431,1489 [M + H]^+^ calcd for C_26_H_22_O_6_ [M + H]^+^ 431,1514.

*(E)-(4-(2,3-dimethoxystyryl)phenyl)(6-methoxybenzofuran-2-yl)methanone (* ***6i*** *):*

Solid light yellow; Yield: 32%; M.p. 145–147°C. ^**1**^**H NMR (300 MHz, CDCl**_**3**_) δ 8.09 (d, *J* = 8.4 Hz, 2H, (2 and 6-ring A)), 7.73 (d, *J* = 8.4 Hz, 2H, (3 and 5-ring A)), 7.66 (d, *J* = 16.5 Hz, 1H, (*E*-styryl)), 7.63 (d, *J* = 8.6 Hz, 1H, (4-benzofuran)), 7.54 (d, *J* = 1.0 Hz, 1H, (3-benzofuran)), 7.24 (d, *J* = 16.5 Hz, 1H, (*E*-styryl)), 7.19–7.09 (m, 2H, (7-benzofuran and 5-ring B)), 7.02 (dd, *J* = 8.7, 2.3 Hz, 1H, (6-ring B)), 6.97–6.88 (m, 2H, (5-benzofuran and 4-ring B)), 3.94 (s, 3 x OMe). ^**13**^**C NMR (75 MHz, CDCl**_**3**_) δ 183.23 (C = O), 161.29 (6-benzofuran), 157.70 (7a-benzofuran), 153.23 (2-benzofuran), 152.16 (3-ring B), 147.36 (2-ring B), 142.17 (4-ring A), 136.32 (1-ring A), 131.03 (1-ring B), 130.06 (2 and 6-ring A), 128.79 (Ar_1_-**C**H = CH-Ar_2_), 126.67 (3 and 5-ring A), 125.84 (Ar_1_-CH = **C**H-Ar_2_), 124.33 (5-ring B), 123.73 (4-benzofuran), 120.51 (3a-benzofuran), 118.06 (6-ring B), 117.06 (3-benzofuran), 114.62 (4-ring B), 112.11 (5-benzofuran), 95.73 (7-benzofuran), 61.33 (OMe), 55.95 (OMe), 55.86 (OMe). Calc. for C_26_H_22_O_5_ [M + H]^+^: 415.1547; Found 415.1552

*(4-(2,5-dimethoxystyryl)phenyl)(6-methoxybenzofuran-2-yl)methanone (* ***6j*** *):*

^**1**^**H NMR (300 MHz, CDCl**_**3**_) δ *E*-isomer: 8.02 (d, *J* = 8.4 Hz, 2H), 7.85 (d, *J* = 8.4 Hz, 2H), 7.63 (d, *J* = 16.1 Hz, 1H, CH = CH_*trans*_), 7.55 (d, *J* = 8.7 Hz, 1H), 7.41 (s, 1H), 7.28 (d, *J* = 16.1 Hz, 1H, CH = CH_*trans*_), 7.25 (t, *J* = 8.4 Hz, 1H), 7.10 (d, *J* = 8.7 Hz, 1H), 6.96–6.91 (m, 1H), 6.61 (d, *J* = 8.4 Hz, 1H), 6.54 (d, *J* = 8.4 Hz, 1H), 3.92 (s, 6H, 2 x OMe), 3.90 (s, 3H, OMe). *Z*-isomer: 8.02 (d, *J* = 8.4 Hz, 2H), 7.85 (d, *J* = 8.4 Hz, 2H), 7.48 (s, 1H), 7.41 (s, 1H), 7.25 (t, *J* = 8.4 Hz, 1H), 7.21 (d, *J* = 12.1 Hz, 1H, CH = CH_*cis*_), 7.10 (d, *J* = 8.4 Hz, 1H), 7.01–6.96 (m, 1H), 6.78 (d, *J* = 12.1 Hz, 1H, CH = CH_*cis*_), 6.61 (d, *J* = 8.4 Hz, 1H), 6.54 (d, *J* = 8.4 Hz, 1H), 3.88 (s, 3H, OMe), 3.63 (s, 6H, 2 x OMe). ^**13**^**C NMR (CDCl**_**3**_**, 75 MHz)**: δ 183.35 (C = O), 161.13 (6-benzofuran), 158.94 (2 and 6-ring B), 157.66 (7a-benzofuran), 152.20 (2-benzofuran), 144.05 (4-ring A), 143.56 (2-ring A), 135.60 (4-ring B), 131.02 (Ar_1_-**C**H = CH-Ar_2_),129.89 (2 and 6-ring A), 129.03 (3 and 5-ring A), 127.89 (3a-benzofuran), 126.32 (Ar_1_-**C**H = CH-Ar_2_), 123.61 (4-benzofuran), 120.48 (1-ring B), 114.46 (3-benzofuran), 114.28 (5-benzofuran), 103.99 (5-ring B) and 103.86 (3-ring B), 95.70 (7-benzofuran), 55.84 (2 x OMe), 55.50 (OMe).

### 4.5. *In vitro* biological assays

#### 4.5.2. Cell lines, culture medium, and treatments.

Biological assays were performed using the non-malignant human colonic epithelial cell line NCM460 and the colorectal cancer cell line SW480. Cells were maintained in Dulbecco’s Modified Eagle Medium (DMEM) supplemented with 5% heat-inactivated fetal bovine serum, which provides the essential nutrients and growth factors required to support cellular viability and optimal conditions. In addition, for all experiments, antibiotic (penicillin/streptomycin) was added at 1% to ensure sterility and prevent microbial contamination. Cultures were incubated at 37 °C in a humidified atmosphere containing 5% CO₂. Before each biological evaluation, all compounds were dissolved in dimethyl sulfoxide (DMSO) to ensure complete solubility in the organic solvent, and the final DMSO concentration in the culture media was strictly kept below 1% to avoid solvent-related cytotoxic effects. In all experimental procedures, parental compounds (molecule 4 and pterostilbene), the equimolar mixture, and the conventional chemotherapeutic agent 5-fluorouracil (5-FU), were incorporated to facilitate comparative analysis. Furthermore, internal untreated controls were included to confirm the non-toxic nature of the solvent control (DMSO) [88–90].

#### 4.5.3. One-dose screening.

The first analysis was performed at one concentration using a modified version of the National Cancer Institute methodology [91,92]. This screening was limited to malignant cells (SW480). Cells were initially seeded into 96-well plates and incubated for 24 hours to facilitate proper adhesion. After this period, experimental compounds were added to the culture media at a final concentration of 100 µM. To assess cell viability, the sulforhodamine B (SRB) assay was employed—a colorimetric technique that measures cellular protein content in adherent populations. For this, cells were fixed using 50% (v/v) trichloroacetic acid (Merck), followed by protein solubilization with 10 mM Tris-base. Cell growth inhibition was calculated by comparing the absorbance values of treated wells to those of the control group exposed only to the vehicle [93].

#### 4.5.4. Cytotoxic and antiproliferative activity through a seven-dose assay.

In the subsequent phase of the study, a seven-dose assay was performed using half-log serial dilutions ranging from 100 µM to 0.10 µM. This phase continues the initial single-dose screening and aims to provide a more in-depth evaluation of the biological activity of the most promising hybrid compounds. Selection criteria for this stage were based on those candidates that exhibited greater than 70% inhibition in the preliminary assay. This test was carried out using the sulforhodamine B (SRB) assay, following the standardized protocol previously described. SW480 and NCM460 cells were included to determine the selectivity indices of the compounds. Control compounds were directly included in this phase without prior screening, given their relevance as reference molecules [94].

#### 4.5.5. Trypan blue exclusion assay.

Cells were plated into 24-well culture plates and allowed to adhere for 24 hours prior to compound exposure. Hybrids **6d** and **6e** were then added to the culture medium at three concentrations (3, 9.5, and 30 µM), selected based on proximity to the GI_50_. Following a 48-hour incubation period, cell viability was assessed by staining with 0.4% trypan blue. After staining, cells were maintained in 1X phosphate-buffered saline (PBS) and examined under an AE31 Elite inverted microscope (Motic China Group Co. Ltd.) using 20X and 40X magnification. Images were acquired from multiple regions within each well using a Moticam ProS5 camera. Viable cells excluded the dye, while non-viable cells were identified by blue staining. All treatments were tested in triplicate.

#### 4.5.6. Mitochondrial and Plasma Membrane Potential Assessment.

To evaluate mitochondrial membrane potential (ΔΨm) and plasma membrane potential (PMP), cells were seeded in 96-well plates and allowed to adhere for 24 hours. Following this, cells were treated with hybrids **6d** and **6e** for 48 hours. After treatment, cells were gently rinsed with PBS (pH 7.4) and incubated with 20 nM 3,3′-Dihexyloxacarbocyanine iodide (DiOC6; Invitrogen, Eugene, OR, USA) for 15 minutes at room temperature in the dark. Imaging was performed in PBS using an AE31E Trinocular Inverted LED Fluorescence Microscope (Motic China Group Co. Ltd.), with excitation at 480 nm and emission at 535 nm.

#### 4.5.7. Cell cycle distribution.

Cells were seeded at 2.5 × 10^5^ cells per well in six-well plates and allowed to adhere for 24 h prior to treatment. After compound exposure, the cultures were incubated for an additional 48 h. At the end of the treatment period, the cells were harvested, fixed by dropwise addition of ice-cold 70% ethanol, and stored overnight at 4°C. The next day, the ethanol was removed, and the cells were washed twice. Residual alcohol was eliminated by an additional wash using PBS. The pellets were then resuspended in 300 μL PBS containing 0.25 mg/mL RNase A (Type I-A, Sigma-Aldrich, Germany) and 0.1 mg/mL propidium iodide (PI). Samples were incubated for 30 min at room temperature in the dark to allow RNA degradation and DNA staining. A minimum of 10,000 events per sample was collected using a FACS Canto II flow cytometer (BD Biosciences, USA). Cell-cycle phase distribution was determined using FlowJo v7.6.2 (Ashland, OR, USA).

#### 4.5.8. Cell migration.

The SW480 cells were seeded and incubated at 37°C with 5% CO_2_. After adherence, the culture medium was removed, the confluent monolayer was scratched with a sterile pipette tip, and the cells were washed with sterile PBS. Subsequently, cells were treated with the compounds (hybrids **6d** and **6e** and the reference drug 5-FU) at their GI_50_ concentrations, maintaining an effective dose per cell. DMSO 0.5% was included as a vehicle control. Cell migration was assessed using an AE31E Trinocular Inverted LED Microscope (Motic China Group Co. Ltd.). Representative wound images were taken at low magnification (10X) from the time of scratch (0h) until 48h after inflicting scratch wounds using a Moticam ProS5 camera. Quantification of wound closure was performed with ImageJ software (NIH, Bethesda, MD, USA) using the formula: %Wound Closure = [(Area_T0_ – Area_TP_)/ Area_T0_] × 100, where Area_T0_ is the initial area immediately after inflicting scratch wounds, and Area_TP_ is the wound area at the indicated time points (24h and 48h).

#### 4.5.9. Statistical analysis.

GraphPad Prism version 8.0.1 (GraphPad Software, San Diego, CA, USA) was used for data analysis. All values are reported as mean ± standard deviation (SD) from two independent technical replicates, each performed in triplicate. Dose–response curves were generated to calculate the following parameters: GI₅₀ (concentration inducing 50% growth inhibition), TGI (concentration at which complete growth arrest occurs), and LC₅₀ (concentration causing a 50% decrease in protein content compared to baseline). The Shapiro–Wilk test was used to assess the normality of the data. Analysis of variance (ANOVA) was conducted to assess statistically significant differences between treatment groups and vehicle control, followed by Tukey’s post hoc test for multiple comparisons. In addition, comparisons among the treatments, the parental compounds (pterostilbene and benzofuran 4), and the reference drug (5-FU) were included. A *p*-value lower than 0.05 was considered statistically significant. Comparisons were made, taking into account the specific treatment, compound concentrations, and cell lines analyzed [95].

#### 4.5.10. Ethical Approval.

There are no human or animal subjects used in this investigation.

#### 4.5.11. Docking studies.

The chemical structure of compounds **6d** and **6e**, as well as the inhibitors or drugs Ac-DEVD-CMK, MMX-9, NSC194598, SCH529074, Talazoparib, Nutlin, Dinaciclib, Abemaciclib, and Etoposide, were used as ligands in the computation approaches. Their 2D structures were drawn using ChemDraw 23.1.1.3 (Cambridge Soft, USA) and saved as MDL Mol files. The Chem3D 23.1 (Cambridge Soft, USA) was used to generate 3D structures of all ligands and energetically minimize them by the MM2 force field. The DS Visualizer 2024 client program was used to convert the data files to the PDB format. AutodockTools were used to parameterize ligand structures, compute Gasteiger partial atomic charges, add full hydrogens, and assign rotatable bonds. The resulting structure was saved in the required format (.pdbqt) for use with AutoDock. Then, AUTOTUTORS in AutoDockTools was used to define all possible flexible torsions of the selected ligands to favor the computed binding to the receptor structure [96]. CDK2 (PDB ID: 4kd1), CDK4 (PDB ID: 7sj3), and CDK6 (PDB ID: 5l2s), caspase-3 (PDB code: 5i9b), caspase-7 (PDB ID: 1f1j), caspase-8 (PDB ID: 3kjn), caspase-9 (PDB ID: 2ar9), PARP-1 (PDB ID: 4und), MDM2 (PDB ID: 4hg7), and TOP2A (PDB ID: 5gwk), and p53 (PDB ID: 1TSR) crystal structures were downloaded from the Protein Data Bank (https://rcsb.org; accessed on 05 Dec 2025), and all bonded ligands, ions, and solvent molecules were manually removed using the DS Visualizer 2.5 program. For the docking studies, the structures of selected proteins were parameterized using AutoDock Tools [97]. In order to facilitate the formation of hydrogen bonds, polar hydrogens were added. AutoDock Vina was used to perform molecular docking, following the default procedures for docking a flexible ligand to a rigid protein. Then, ligands were centered at the binding site located into the binding cavity of the CDK2 (x = 55.037, y = 77.945, z = 27.554), CDK4 (x = 16.7, y = −37.38, z = 11.721), CDK6 (x = 24.431, y = 34.548, z = −9.469), caspase-3 (x = 2.584, y = −8.037, z = −18.547), caspase-7 (x = 37.955, y = 23.117, z = −2.671), caspase-8 (x = −8.437 y = 33.08, z = 36.984), caspase-9 (x = 20.595, y 36.897, z = −12.993), PARP-1 (chain A, x = 2.950, y = 67.758, z = 189.704), MDM2 (x = −23.549, y = 11.143, z = −11.961), TOP2A (x = 23.871, y = −42.291z = −57.273), p53 (x = 58.715, y = 27.197, z = 79.739). In detail, docking studies involved a grid box, which was identified by using Autodock Vina 1.1.2, and exhaustiveness was 20 for each protein-compound pair [97]. Catalytic active site was surrounded by a docking grid of 36 × 36 × 36 Å (for CDK2, CDK4, PARP-1), 40 × 40 × 40 Å (for CDK6, Caspase-3/8, TOP2A), 42 × 42 × 42 Å (for Caspase-7), and 38 × 38 × 38 Å (for caspase 9), 40 × 46 × 40 Å (for MDM2, P53) with a grid spacing of 1Å. Ligand-binding affinities (in kcal/mol) were estimated by AutoDock Vina and ranked based on the free energy binding theory. Then, docking solutions were graphically inspected by using the DS Visualizer 2024 client to provide a 2D-ligand interaction plot, while ribbon surface representation of the 3D model was explored by using The PyMOL Molecular Graphics System Version 2.3.2, Schrodinger, LLC (2025).

#### 4.5.12. ADME-*tox* modelling studies.

The pharmacokinetic profile of the lead compounds **6d** and **6e** was evaluated using SwissADME, an open-source cheminformatics platform [98]. Key biopharmaceutical parameters assessed included the fraction of sp³-hybridized carbon atoms, number of aromatic/heteroaromatic rings, human serum albumin binding affinity (logK_HSA_), predicted intestinal permeability (Caco-2 and MDCK models), topological polar surface area (TPSA), molecular weight, number of rotatable bonds, lipophilicity (logP_o/w_), and gastrointestinal absorption (GI). In addition, substructural alerts, such as the PanAssay Interference Compound (PAINS), were screened. The toxicological profile of **6d** and **6e** was further predicted using a suite of freely available computational tools, including SwissADME, OSIRIS, TEST (v5.1.2), ProTox-II, Pred-hERG, pkCSM, ToxTree, and ADMET-SAR [98–105].

## Supporting information

S1. File Compound 2. The physicochemical properties, spectral characterization details and copy of ^1^H NMR and ^13^C NMR of *2-bromo-1-(4-bromophenyl)ethan-1-one* (***2***).
(PDF)

S2. File Compound 4. The physicochemical properties, spectral characterization details and copy of ^1^H NMR and ^13^C NMR of *(4-bromophenyl)(6-methoxybenzofuran-2-yl)methanone* (***4***).
(PDF)

S3. File Compound 6a. The physicochemical properties, spectral characterization details and copy of ^1^H NMR, ^13^C NMR and mass spectra of *(E)-(4-(2,4-dimethoxystyryl)phenyl)(6-methoxybenzofuran-2-yl)methanone* (***6a***).
(PDF)

S4. File Compound 6b. The physicochemical properties, spectral characterization details and copy of ^1^H NMR, ^13^C NMR and mass spectra of *(E)-(4-(2,5-dimethoxystyryl)phenyl)(6-methoxybenzofuran-2-yl)methanone* (***6b***).
(PDF)

S5. File Compound 6c. The physicochemical properties, spectral characterization details and copy of ^1^H NMR, ^13^C NMR and mass spectra of *(E)-(4-(3,5-dimethoxystyryl)phenyl)(6-methoxybenzofuran-2-yl)methanone* (***6c***).
(PDF)

S6. File Compound 6d. The physicochemical properties, spectral characterization details and copy of ^1^H NMR, ^13^C NMR and mass spectra of *(E)-(4-(3,4-dimethoxystyryl)phenyl)(6-methoxybenzofuran-2-yl)methanone* (***6d***).
(PDF)

S7. File Compound 6e. The physicochemical properties, spectral characterization details and copy of ^1^H NMR, ^13^C NMR and mass spectra of *(E)-(4-(4-hydroxy-3-methoxystyryl)phenyl)(6-methoxybenzofuran-2-yl)methanone* (***6e***).
(PDF)

S8. File Compound 6f. The physicochemical properties, spectral characterization details and copy of ^1^H NMR, ^13^C NMR and mass spectra of *(E)-(6-methoxybenzofuran-2-yl)(4-(2,4,5-trimethoxystyryl)phenyl)methanone* (***6f***).
(PDF)

S9. File Compound 6g. The physicochemical properties, spectral characterization details and copy of ^1^H NMR, ^13^C NMR and mass spectra of *(E)-(6-methoxybenzofuran-2-yl)(4-(3,4,5-trimethoxystyryl)phenyl)methanone* (***6g***).
(PDF)

S10. File Compound 6h. The physicochemical properties, spectral characterization details and copy of ^1^H NMR, ^13^C NMR and mass spectra of *(E)-(4-(4-hydroxy-3,5-dimethoxystyryl)phenyl)(6-methoxybenzofuran-2-yl)methanone* (***6h***).
(PDF)

S11. File Compound 6i. The physicochemical properties, spectral characterization details and copy of ^1^H NMR, ^13^C NMR and mass spectra of *(E)-(4-(2,3-dimethoxystyryl)phenyl)(6-methoxybenzofuran-2-yl)methanone* (***6i***).
(PDF)

S12. File Compound 6j. Spectral characterization details and copy of ^1^H NMR and ^13^C NMR of *(4-(2,6-dimethoxystyryl)phenyl)(6-methoxybenzofuran-2-yl)methanone (6j)*.
(PDF)

## Abbreviations

5-FU: 5-Fluorouracil
CRC: Colorectal cancer
DIOC6: 3,3’-dihexyloxacarbocyanine iodide
DMSO: Dimethylsulfoxide
FDA: Food and Drug Administration
GI50: Growth inhibition of 50%
IC50: Half maximal inhibitory concentration
LC50: Lethal concentration of 50%
MLT: Melatonin
MMP: Matrix metalloproteinase
N.I.: Not inhibition
N.L.: Non-lethal
NCI: National Cancer Institute
PBS: Phosphate-buffered saline
PIH: Pyridoxal isonicotinoyl hydrazone
PMP: Plasma membrane potential
PT: Pterostilbene
RIH: Resveratrol- isonicotinoyl hydrazone
SAR: Structure-Activity Relationship
SI: selectivity index
TGI: Total growth inhibition
Ψm: mitochondrial membrane potential
