# Supplementary material for: Novel benzofuran/pterostilbene hybrids trigger programmed cell death and impair migration in CRC cells
Source: PLoS One. 2026 Apr 13;21(4):e0344602. doi: 10.1371/journal.pone.0344602 (PMC13075696; doi:10.1371/journal.pone.0344602)
Supplement: S1. File — The physicochemical properties, spectral characterization details and copy of 1H NMR and 13C NMR of 2-bromo-1-(4-bromophenyl)ethan-1-one (2). (PDF) [file pone.0344602.s001.pdf]

S1- The physicochemical properties, spectral characterization details and copy of ^{1}H NMR and ^{13}C NMR of *2-bromo-1-(4-bromophenyl)ethan-1-one (**2**)*.

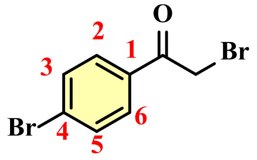

90% yield. White solid, m.p. 98-102°C; **^{1}H-NMR (300 MHz, $CDCl_3$)** δ 7.90 (d, *J* = 8.6 Hz, 2H, (2 and 6)), 7.69 (d, *J* = 8.6 Hz, 2H, (3 and 5)), 4.45 (s, 2H (2)). **^{13}C-NMR (75 MHz, $CDCl_3$)** δ 190.47 (C=O), 132.63 (1’), 132.27 (3 and 5), 130.47 (2 and 6), 129.37 (4), 30.46 (-CH_2-Br).
