# Supplementary material for: Novel benzofuran/pterostilbene hybrids trigger programmed cell death and impair migration in CRC cells
Source: PLoS One. 2026 Apr 13;21(4):e0344602. doi: 10.1371/journal.pone.0344602 (PMC13075696; doi:10.1371/journal.pone.0344602)
Supplement: S2. File — The physicochemical properties, spectral characterization details and copy of 1H NMR and 13C NMR of (4-bromophenyl)(6-methoxybenzofuran-2-yl)methanone (4). (PDF) [file pone.0344602.s002.pdf]

S2- The physicochemical properties, spectral characterization details and copy of ^{1}H NMR and ^{13}C NMR of *(4-bromophenyl)(6-methoxybenzofuran-2-yl)methanone (**4**)*.

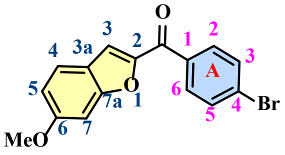

Light yellow solid; Yield: 90%; M.p. 200-202 °C; **^{1}H NMR (600 MHz, $CDCl_3$)** δ 7.92 (d, *J* = 8.6 Hz, 2H, (2 and 6)), 7.68 (d, *J* = 8.5 Hz, 2H, (3 and 5)), 7.60 (d, *J* = 8.7 Hz, 1H, (4-benzofuran)), 7.49 (s, 1H, (3-benzofuran)), 7.08 (d, *J* = 2.2 Hz, 1H, (7-benzofuran)), 6.99 (dd, *J* = 8.7, 2.2 Hz, 1H, (5-benzofuran)). **^{13}C NMR (75 MHz, $CDCl_3$)** δ 182.66 (C=O), 161.46 (6-benzofuran), 157.77 (7a-benzofuran), 151.65 (2-benzofuran), 136.21 (1), 131.85 (3 and 5)), 130.91 (2 and 6), 127.74 (4), 123.76 (3a-benzofuran), 120.30 (4-benzofuran), 117.40 (2-benzofuran), 114.79 (5-benzofuran), 95.61 (7-benzofuran), 55.81 (OMe).

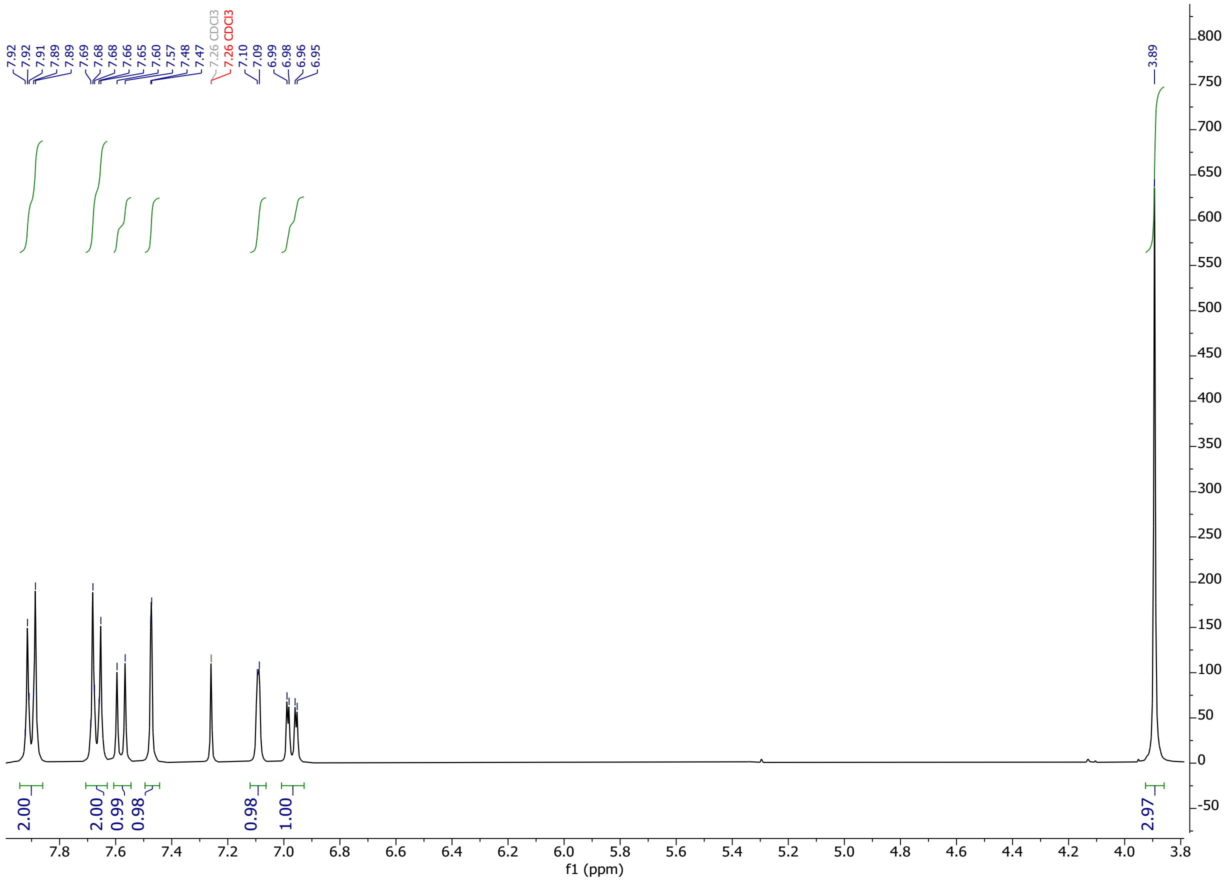

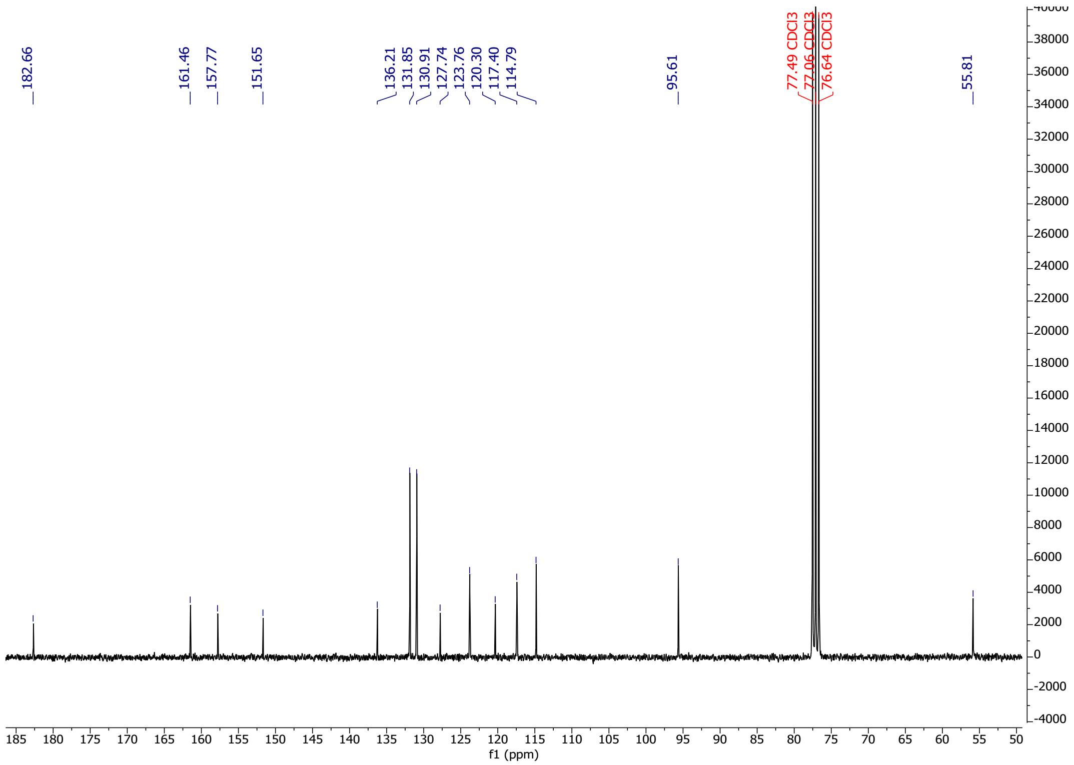

182.66
161.46
157.77
151.65
136.21
131.85
130.91
127.74
123.76
120.30
117.40
114.79
95.61
77.49 CDCl3
77.06 CDCl3
76.64 CDCl3
55.81
f1 (ppm)
