# Supplementary material for: Novel benzofuran/pterostilbene hybrids trigger programmed cell death and impair migration in CRC cells
Source: PLoS One. 2026 Apr 13;21(4):e0344602. doi: 10.1371/journal.pone.0344602 (PMC13075696; doi:10.1371/journal.pone.0344602)
Supplement: S5. File — The physicochemical properties, spectral characterization details and copy of 1H NMR, 13C NMR and mass spectra of (E)-(4-(3,5-dimethoxystyryl)phenyl)(6-methoxybenzofuran-2-yl)methanone (6c). (PDF) [file pone.0344602.s005.pdf]

S5- The physicochemical properties, spectral characterization details and copy of ^{1}H NMR, ^{13}C NMR and mass spectra of *(E)-(4-(3,5-dimethoxystyryl)phenyl)(6-methoxybenzofuran-2-yl)methanone (**6c**)*.

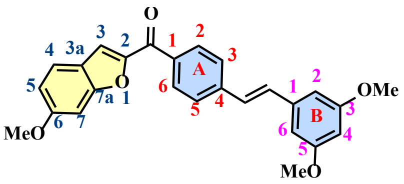

^{1}H NMR (300 MHz, CDCl$_3$) δ 8.04 (d, J = 8.2 Hz, 2H (2 and 6-ring A)), 7.64 (d, J = 8.6 Hz, 2H (3 and 5-ring A)), 7.58 (d, J = 8.7 Hz, 1H (4-benzofuran)), 7.49 (s_{app}, 1H (3-benzofuran)), 7.19 (d, J = 16.3 Hz, 1H (*E*-styryl)), 7.14 (d, J = 16.4 Hz, 1H, (*E*-styryl)), 7.11 (s_{app}, 1H (7-benzofuran)), 6.97 (dd, J = 8.7, 2.2 Hz, 1H (5-benzofuran)), 6.71 (d, J = 2.3 Hz, 2H (2 and 6-ring B)), 6.44 (t, J = 2.2 Hz, 1H (4-ring B)), 3.89 (s, OMe), 3.85 (s, 2 x OMe). **^{13}C NMR (75 MHz, CDCl$_3$)** δ 183.23 (C=O),161.33 (6-benzofuran), 161.17 (3 and 5- ring B), 157.74 (7a-benzofuran), 152.12 (2-benzofuran), 141.63 (4-ring A), 138.83 (1-ring B), 136.46 (1-ring A), 131.53 (Ar_1-**<u>C</u>**H=CH-Ar_2), 130.09 (2 and 6-ring A), 128.12 (Ar_1-CH=**<u>C</u>**H-Ar_2), 126.62 (3 and 5-ring A), 123.76 (3a-benzofuran), 120.51 (4-benzofuran), 117.12 (3-benzofuran), 114.66 (5-benzofuran), 105.02 (2 and 6-ring B), 100.71 (4-ring B), 95.75 (7-benzofuran), 55.89 (OMe), 55.55 (2 x OMe). ESI-MS(*m/z*): 415,1540 $[M+H]^+$ calcd for $C_{26}H_{22}O_5$ $[M+H]^+$ 415,1561.

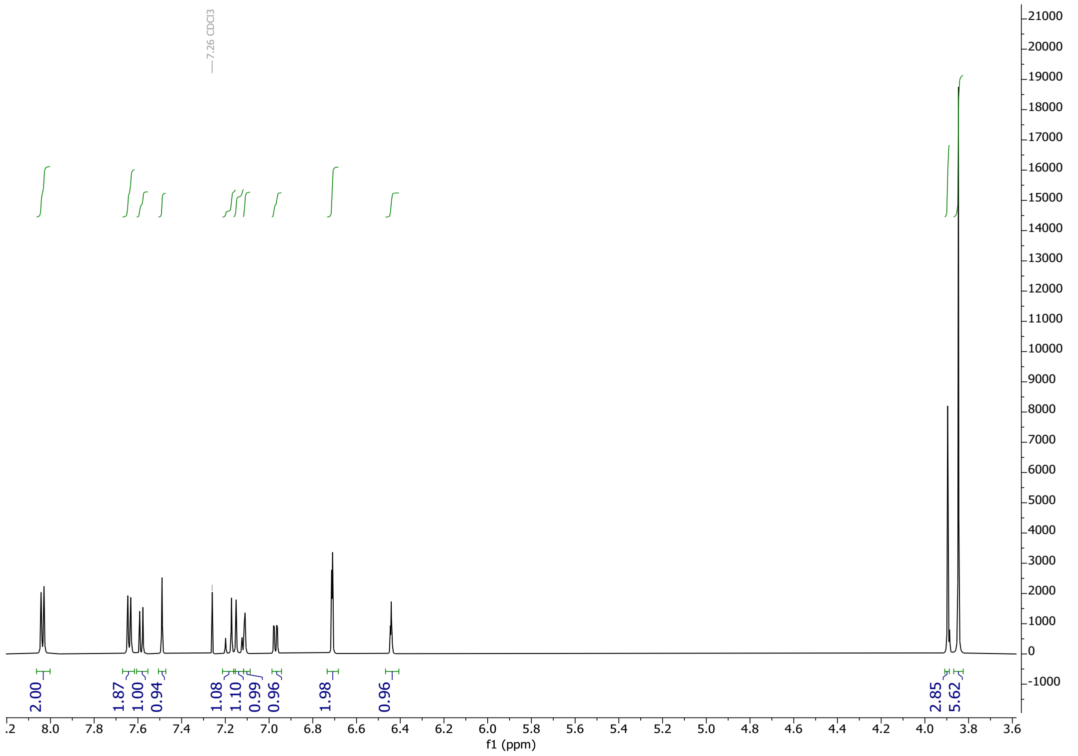

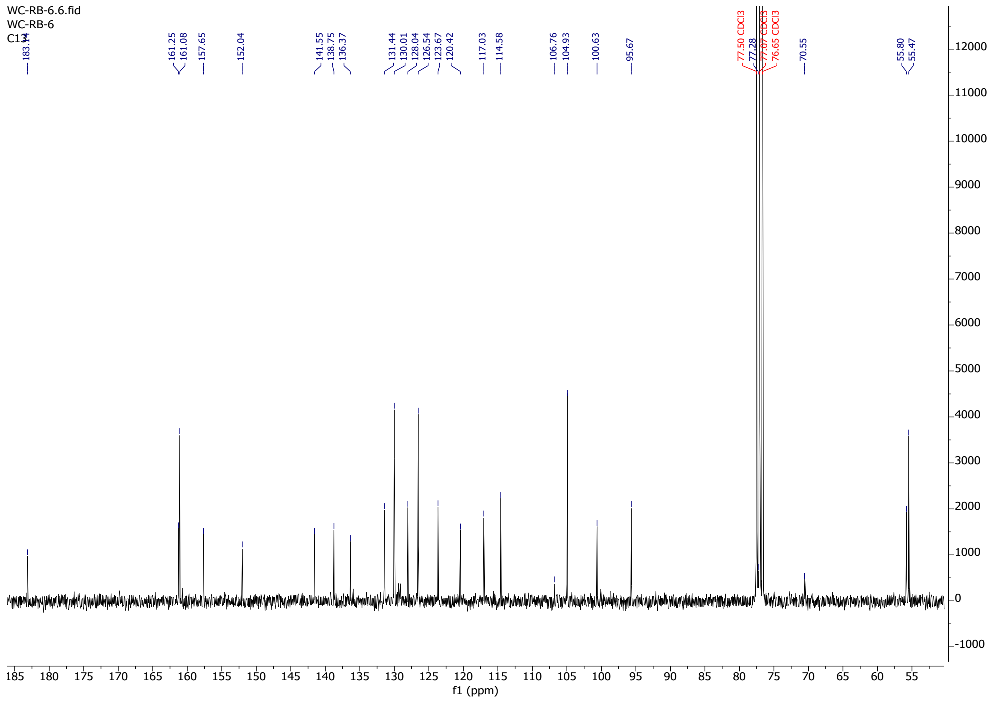
WC-RB-6.6.fid
WC-RB-6
C13
183.11
161.25
161.08
157.65
152.04
141.55
138.75
136.37
131.44
130.01
128.04
126.54
123.67
120.42
117.03
114.58
106.76
104.93
100.63
95.67
77.50 CDCl3
77.28
77.07 CDCl3
76.65 CDCl3
70.55
55.80
55.47
f1 (ppm)

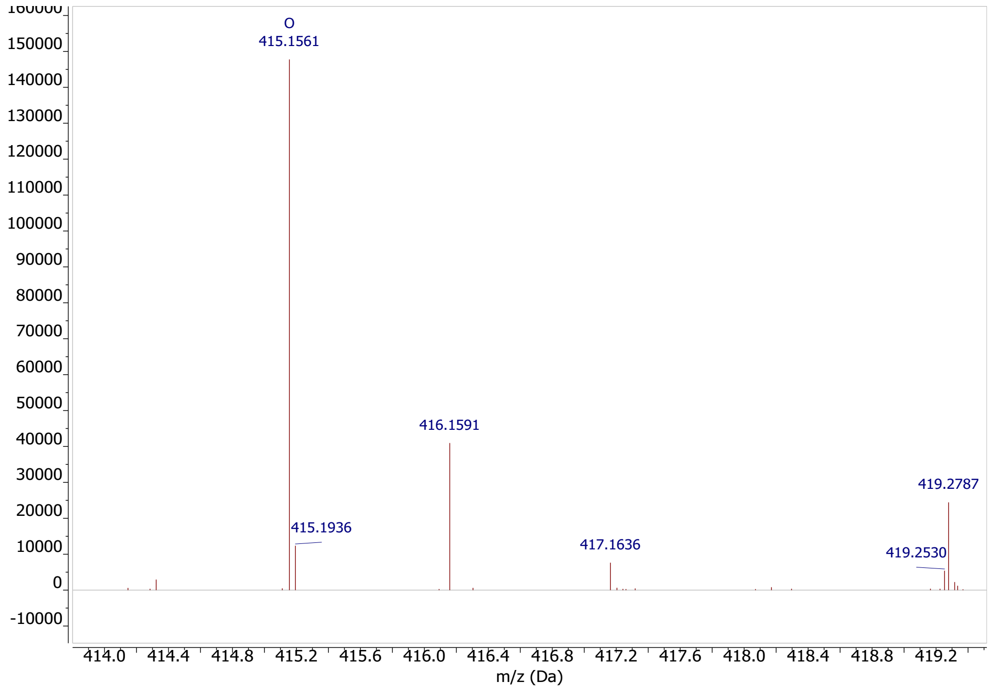
O
415.1561
415.1936
416.1591
417.1636
419.2530
419.2787
m/z (Da)
