# Supplementary material for: Novel benzofuran/pterostilbene hybrids trigger programmed cell death and impair migration in CRC cells
Source: PLoS One. 2026 Apr 13;21(4):e0344602. doi: 10.1371/journal.pone.0344602 (PMC13075696; doi:10.1371/journal.pone.0344602)
Supplement: S6. File — The physicochemical properties, spectral characterization details and copy of 1H NMR, 13C NMR and mass spectra of (E)-(4-(3,4-dimethoxystyryl)phenyl)(6-methoxybenzofuran-2-yl)methanone (6d). (PDF) [file pone.0344602.s006.pdf]

S6- The physicochemical properties, spectral characterization details and copy of ^{1}H NMR, ^{13}C NMR and mass spectra of *(E)-(4-(3,4-dimethoxystyryl)phenyl)(6-methoxybenzofuran-2-yl)methanone* ***(6d)***.

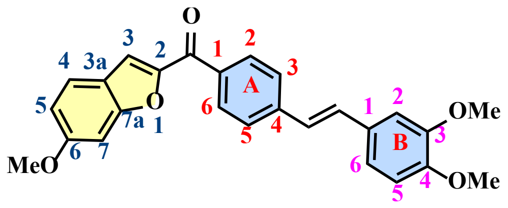

^{1}H NMR (300 MHz, CDCl$_3$) δ 8.04 (d, J = 8.3 Hz, 2H, (2 and 6-ring A)), 7.63 (d, J = 8.3 Hz, 2H, (3 and 5-ring A)), 7.59 (d, J = 8.6 Hz, 1H, (4-benzofuran)), 7.50 (s$_{app}$, , 1H, (3-benzofuran)), 7.21 (d, J = 16.2 Hz, 1H, (*E*-styryl)), 7.12 (d, J = 2.3 Hz, 2H, (7-benzofuran)), 7.12-7.09 (m, 2H, (2 and 6-ring B)), 7.04 (d, J = 16.2 Hz, 1H, (*E*-styryl)), 6.97 (dd, J = 8.7, 2.2 Hz, 1H, (5-benzofuran)), 6.89 (d, J = 8.7 Hz, 1H, (5-ring B)), 3.97 (s, OMe), 3.92 (s, OMe), 3.90 (s, OMe). **^{13}C NMR (75 MHz, CDCl$_3$)** δ 183.27 (C=O), 161.32 (6-benzofuran), 157.73 (7a-benzofuran), 152.19 (2-benzofuran), 149.66 (3-ring B), 149.34 (4-ring B), 142.16 (4-ring A), 136.04 (1-ring A), 131.42 (1-ring B), 130.15 (2 and 6-ring A), 129.96 (Ar$_1$-**C**H=CH-Ar$_2$), 126.30 (3 and 5-ring A), 125.68 (Ar$_1$-CH=**C**H-Ar$_2$), 123.75 (3a-benzofuran), 120.68 (4-benzofuran), 120.54 (6-ring B), 117.05 (3-benzofuran), 114.65 (5-benzofuran), 111.34 (5-ring B), 109.00 (2-ring B), 95.78 (7-benzofuran), 56.11 (OMe), 56.06 (OMe), 55.91 (OMe). ESI-MS(*m/z*): 415,1540 $[M+H]^+$ calcd for $C_{26}H_{22}O_5$ $[M+H]^+$ 415,1559.

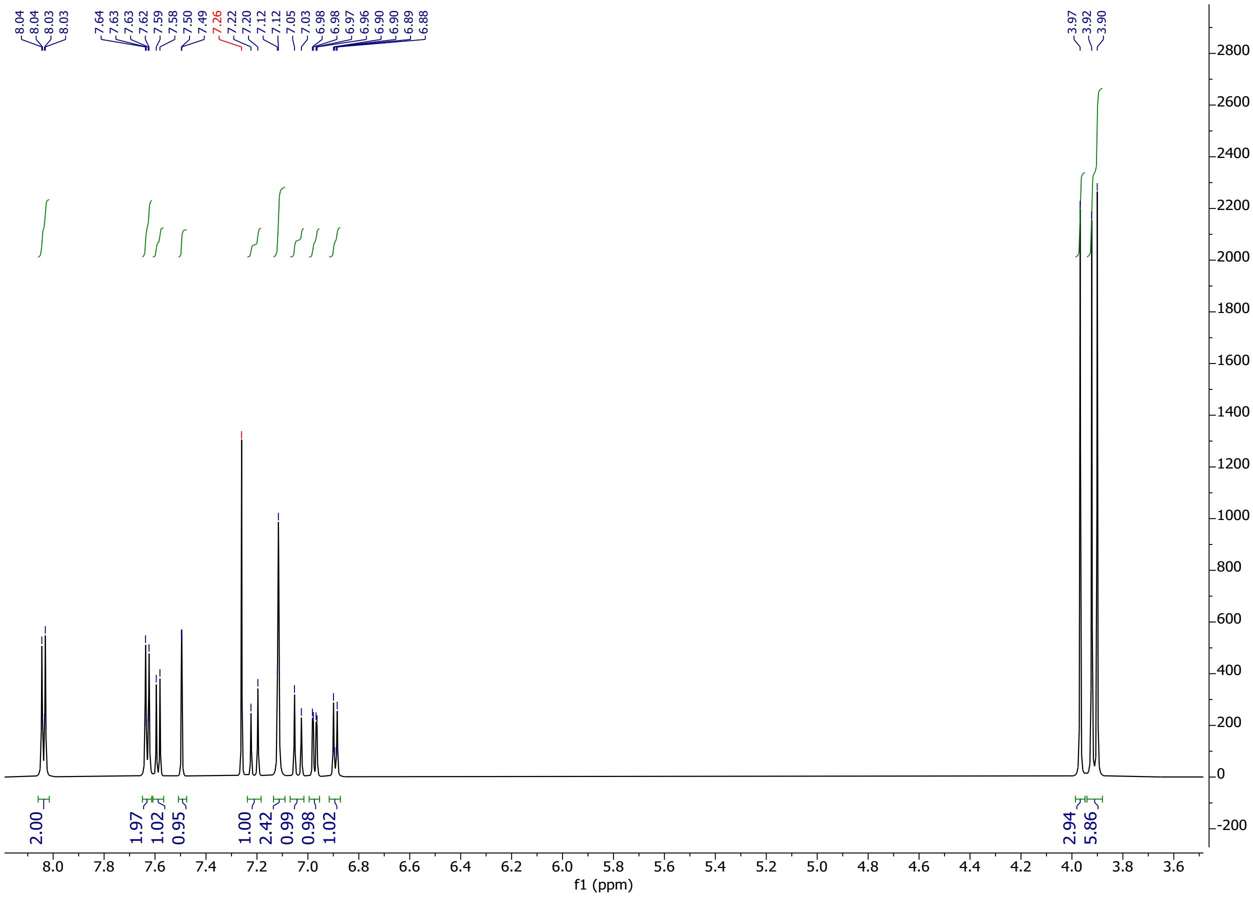

8.04
8.04
8.03
8.03
7.64
7.63
7.63
7.62
7.59
7.58
7.50
7.49
7.26
7.22
7.20
7.12
7.05
7.03
6.98
6.98
6.97
6.96
6.90
6.90
6.89
6.88
3.97
3.92
3.90
2.00
1.97
1.02
0.95
1.00
2.42
0.99
0.98
1.02
2.94
5.86
f1 (ppm)

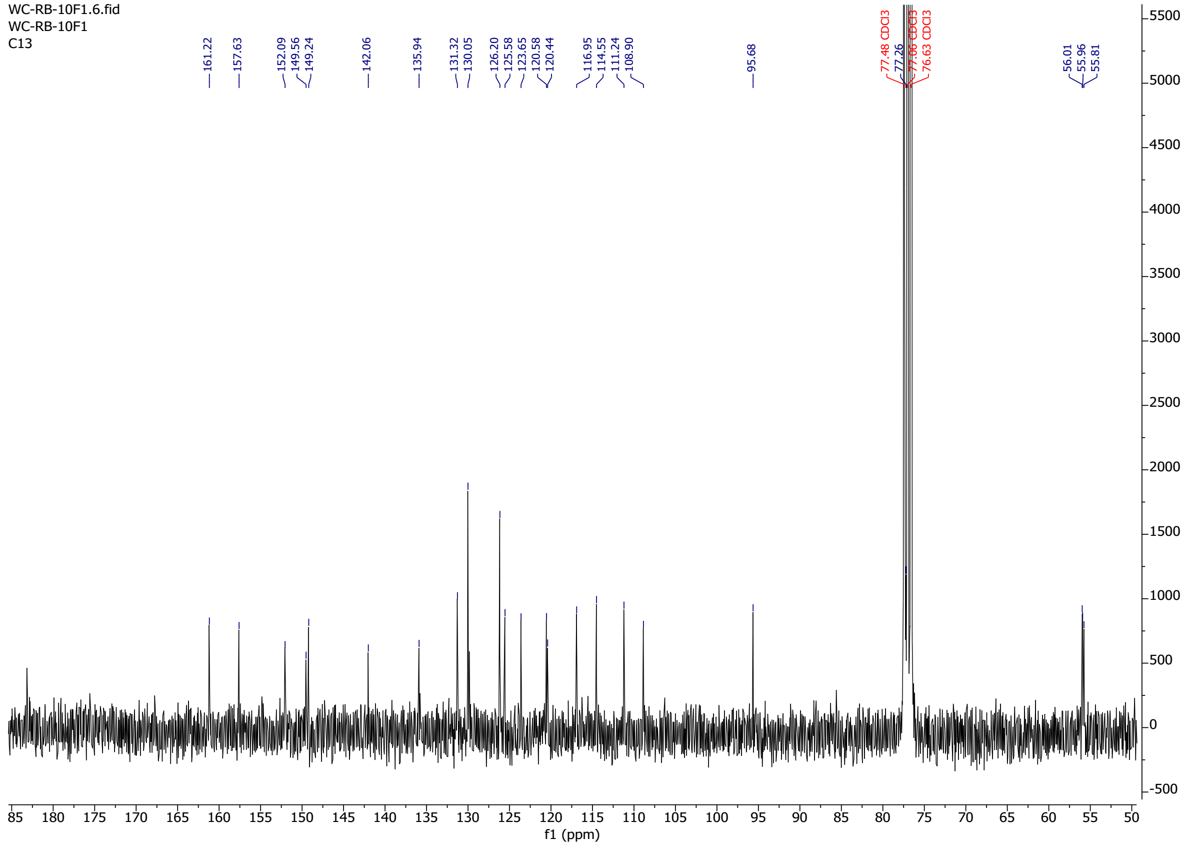

WC-RB-10F1.6.fid
WC-RB-10F1
C13
161.22
157.63
152.09
149.56
149.24
142.06
135.94
131.32
130.05
126.20
125.58
123.65
120.58
120.44
116.95
114.55
111.24
108.90
95.68
77.48 CDCl3
77.26
77.06 CDCl3
76.63 CDCl3
56.01
55.96
55.81
f1 (ppm)

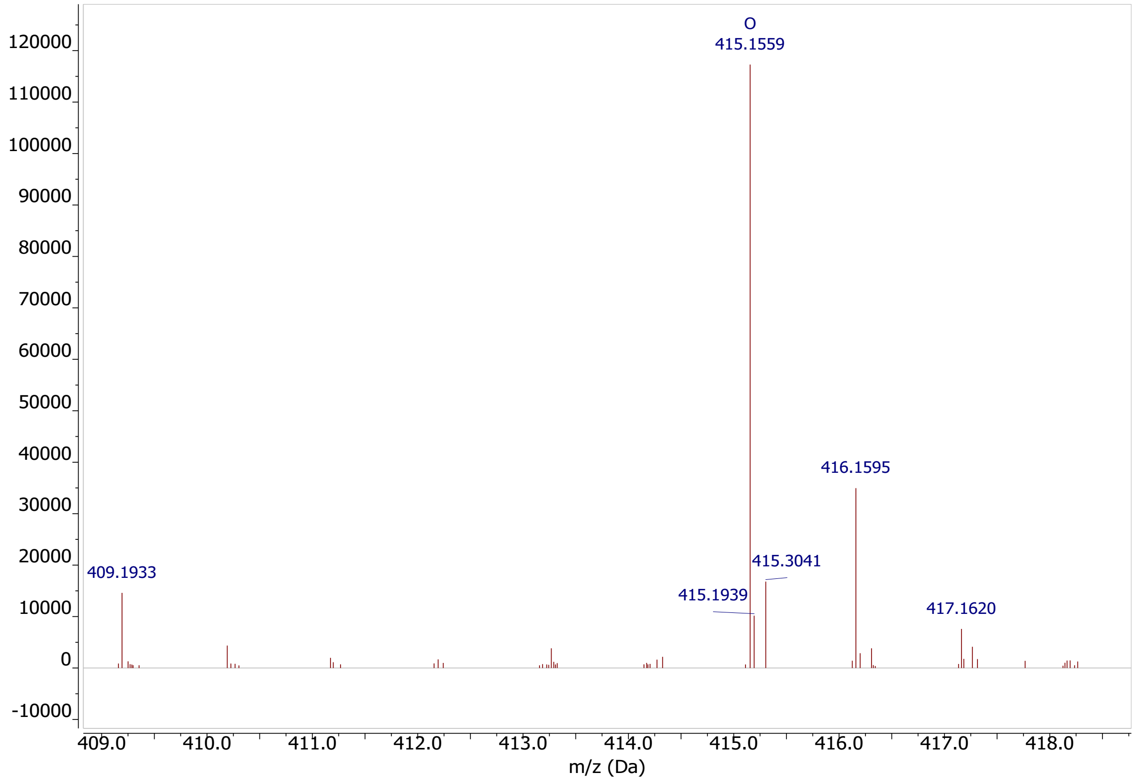

O
415.1559
416.1595
415.3041
415.1939
409.1933
417.1620
120000
110000
100000
90000
80000
70000
60000
50000
40000
30000
20000
10000
0
-10000
409.0
410.0
411.0
412.0
413.0
414.0
415.0
416.0
417.0
418.0
m/z (Da)
