# Supplementary material for: Novel benzofuran/pterostilbene hybrids trigger programmed cell death and impair migration in CRC cells
Source: PLoS One. 2026 Apr 13;21(4):e0344602. doi: 10.1371/journal.pone.0344602 (PMC13075696; doi:10.1371/journal.pone.0344602)
Supplement: S9. File — The physicochemical properties, spectral characterization details and copy of 1H NMR, 13C NMR and mass spectra of (E)-(6-methoxybenzofuran-2-yl)(4-(3,4,5-trimethoxystyryl)phenyl)methanone (6g). (PDF) [file pone.0344602.s009.pdf]

S9- The physicochemical properties, spectral characterization details and copy of ^{1}H NMR, ^{13}C NMR and mass spectra of *(E)-(6-methoxybenzofuran-2-yl)(4-(3,4,5-trimethoxystyryl)phenyl)methanone* ***(6g)***.

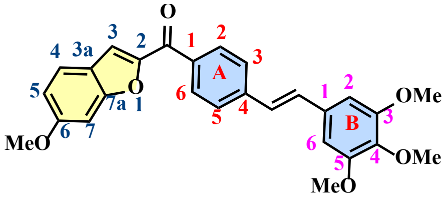

^{1}H NMR (300 MHz, $CDCl_3$) δ 8.04 (d, J = 8.3 Hz, 2H, (2 and 6-ring A)), 7.64 (d, J = 8.5 Hz, 2H, (3 and 5-ring A)), 7.59 (d, J = 8.6 Hz, 1H, (4-benzofuran)), 7.50 (s, 1H, (3-benzofuran)), 7.19 (d, J = 16.2 Hz, 1H, (*E*-styryl)), 7.11 (s, 1H, (7-benzofuran)), 7.07 (d, J = 16.2 Hz, 1H, (*E*-styryl)), 6.97 (dd, J = 8.7, 2.2 Hz, 1H, (5-benzofuran)), 6.79 (s, 2H, (2 and 6-ring B)), 3.94 (2 x OMe), 3.90 (OMe), 3.89 (OMe). **^{13}C NMR (75 MHz, $CDCl_3$)** δ 183.10 (C=O), 161.23 (6-benzofuran), 157.64 (7a-benzofuran), 153.50 (3 and 5-ring B), 152.03 (2-benzofuran), 141.64 (4-ring A), 138.53 (4-ring B), 136.22 (1 ring A), 132.46 (1-ring B), 131.42 (Ar_1-**C**H=CH-Ar_2), 130.03 (2 and 6 ring A), 126.97 (Ar_1-CH=**C**H-Ar_2), 126.36 (3 and 5 ring A), 123.65 (4-benzofuran), 120.41 (3a-benzofuran), 116.99 (3-benzofuran), 114.56 (5-benzofuran), 103.94 (2 and 6-ring B), 95.67 (7-benzofuran), 61.04 (OMe), 56.21 (2 x OMe), 55.80 (OMe). ESI-MS(*m/z*): 445,1646 $[M+H]^+$ calcd for $C_{27}H_{24}O_6$ $[M+H]^+$ 445,1663.

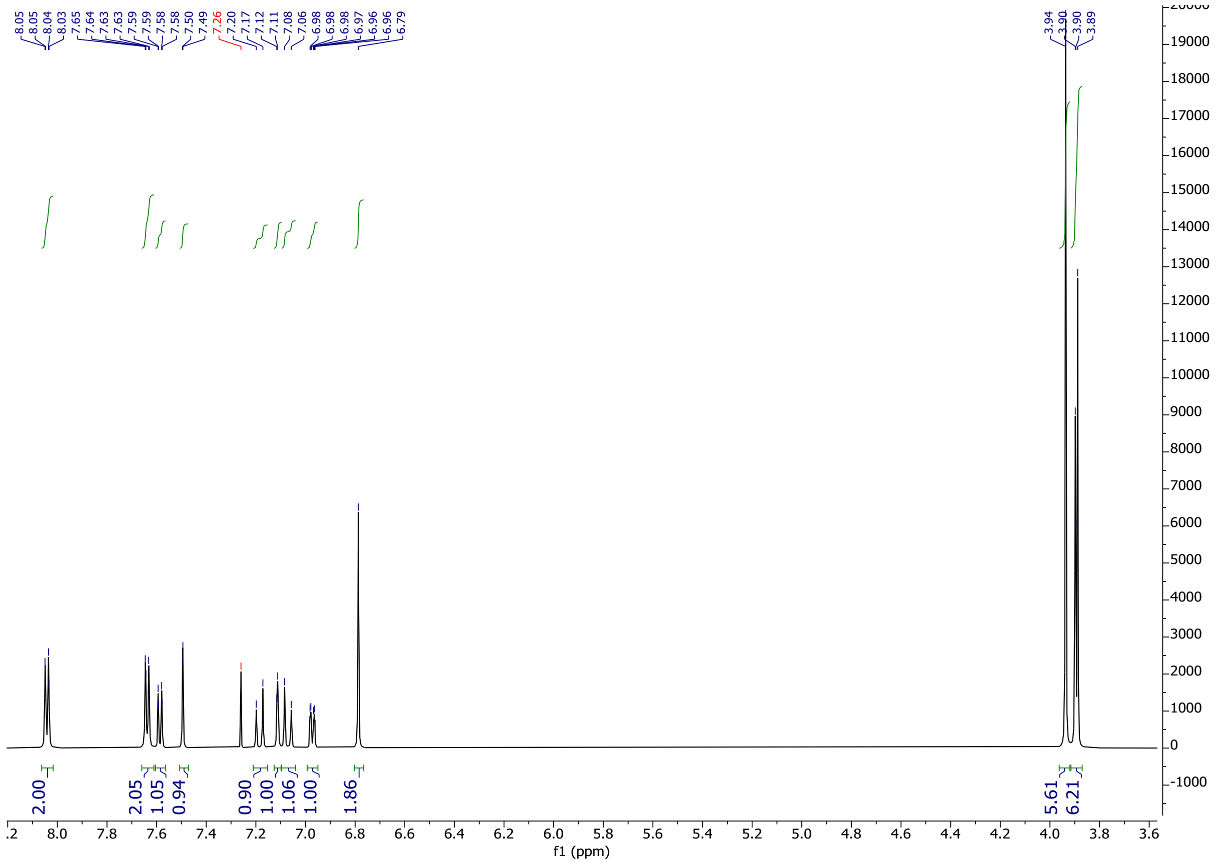

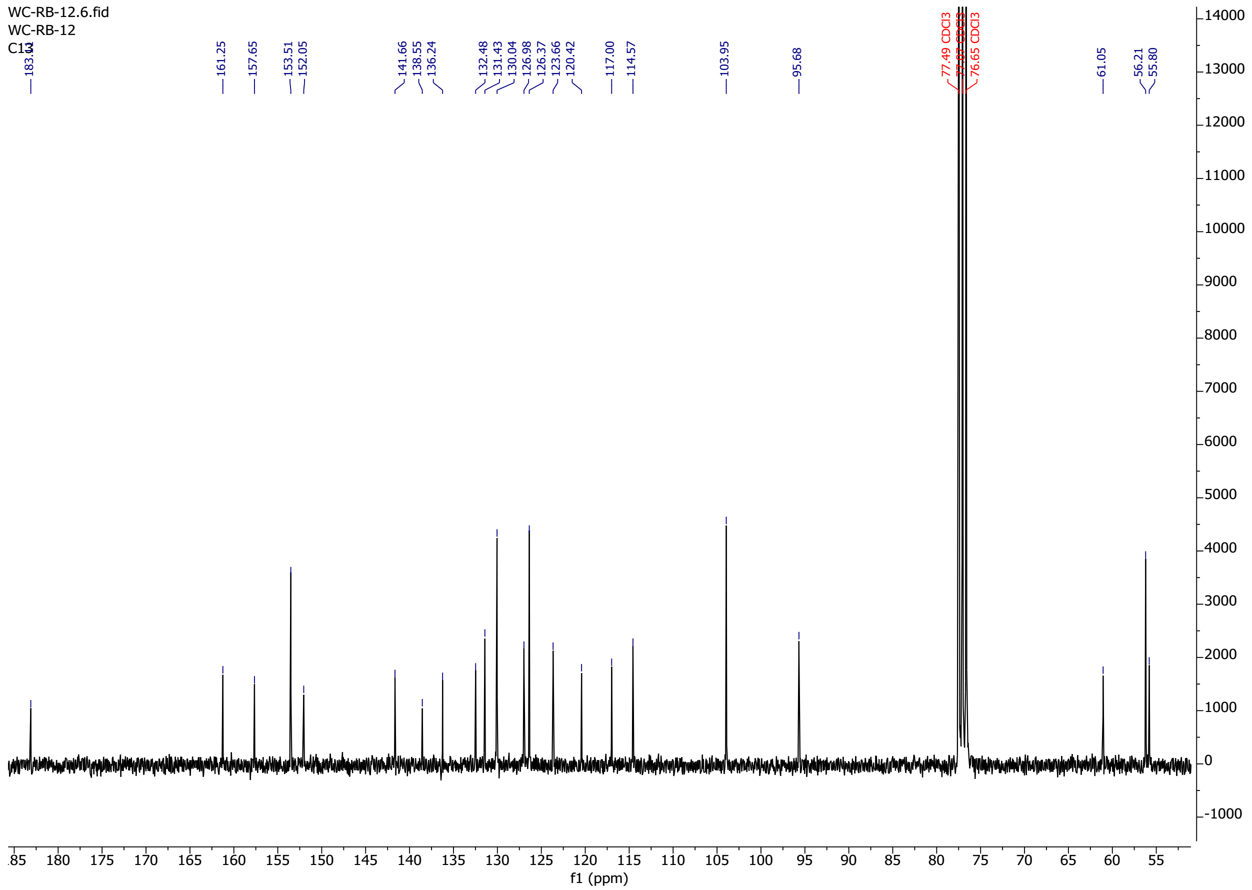

WC-RB-12.6.fid
WC-RB-12
C13
183.3
161.25
157.65
153.51
152.05
141.66
138.55
136.24
132.48
131.43
130.04
126.98
126.37
123.66
120.42
117.00
114.57
103.95
95.68
77.49 CDCl3
76.65 CDCl3
61.05
56.21
55.80
f1 (ppm)

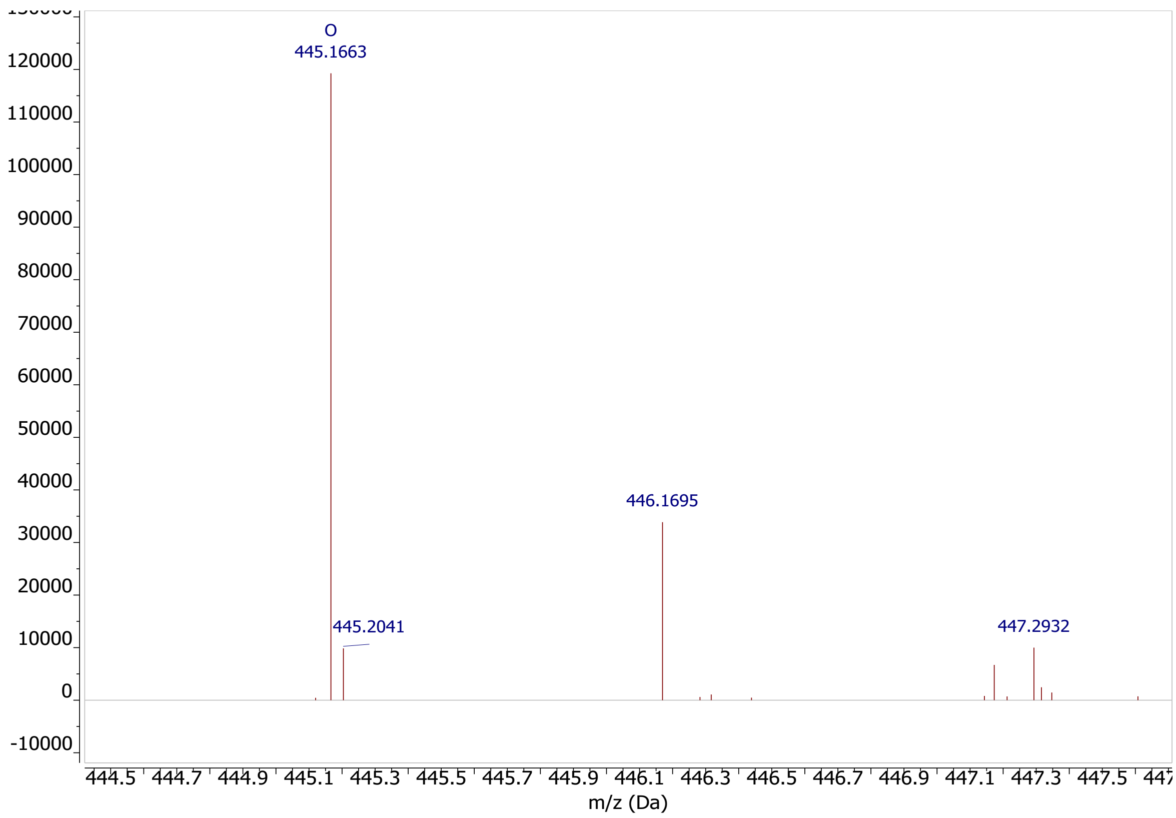

O
445.1663
445.2041
446.1695
447.2932
m/z (Da)
