# Supplementary figures and images for: Novel benzofuran/pterostilbene hybrids trigger programmed cell death and impair migration in CRC cells
Source: PLoS One. 2026 Apr 13;21(4):e0344602. doi: 10.1371/journal.pone.0344602 (PMC13075696; doi:10.1371/journal.pone.0344602)

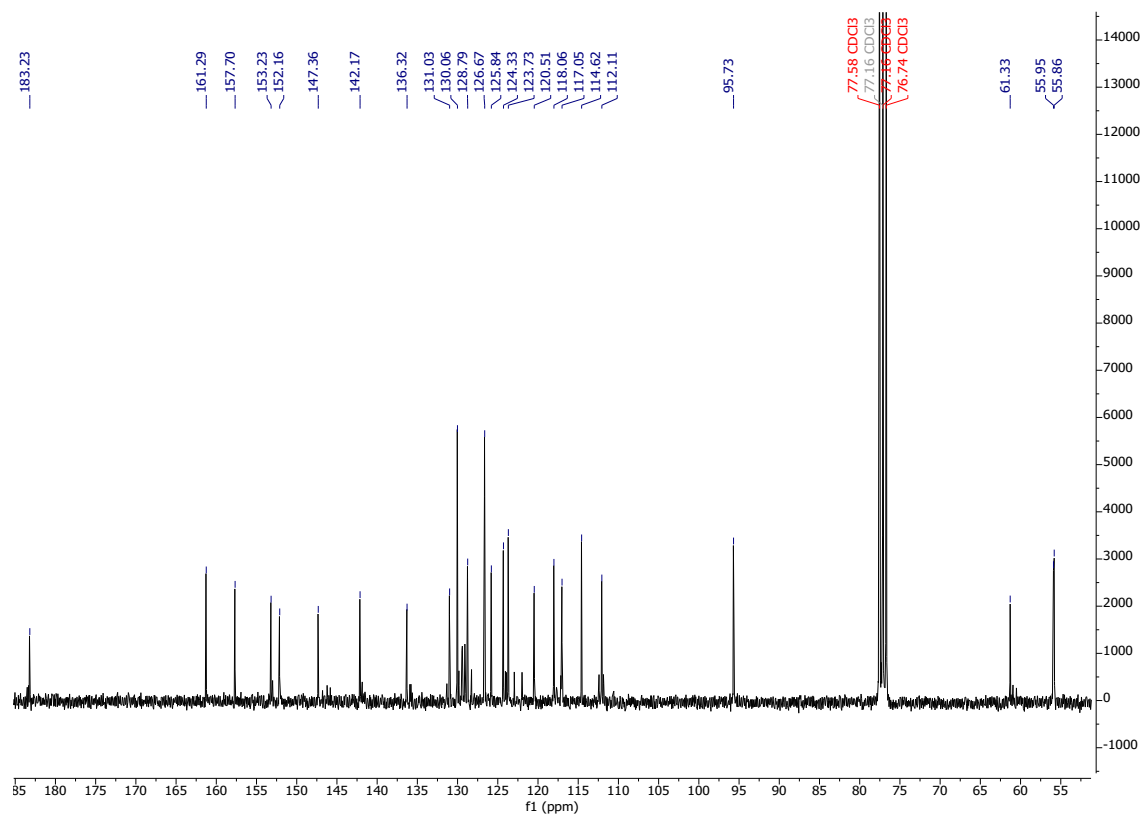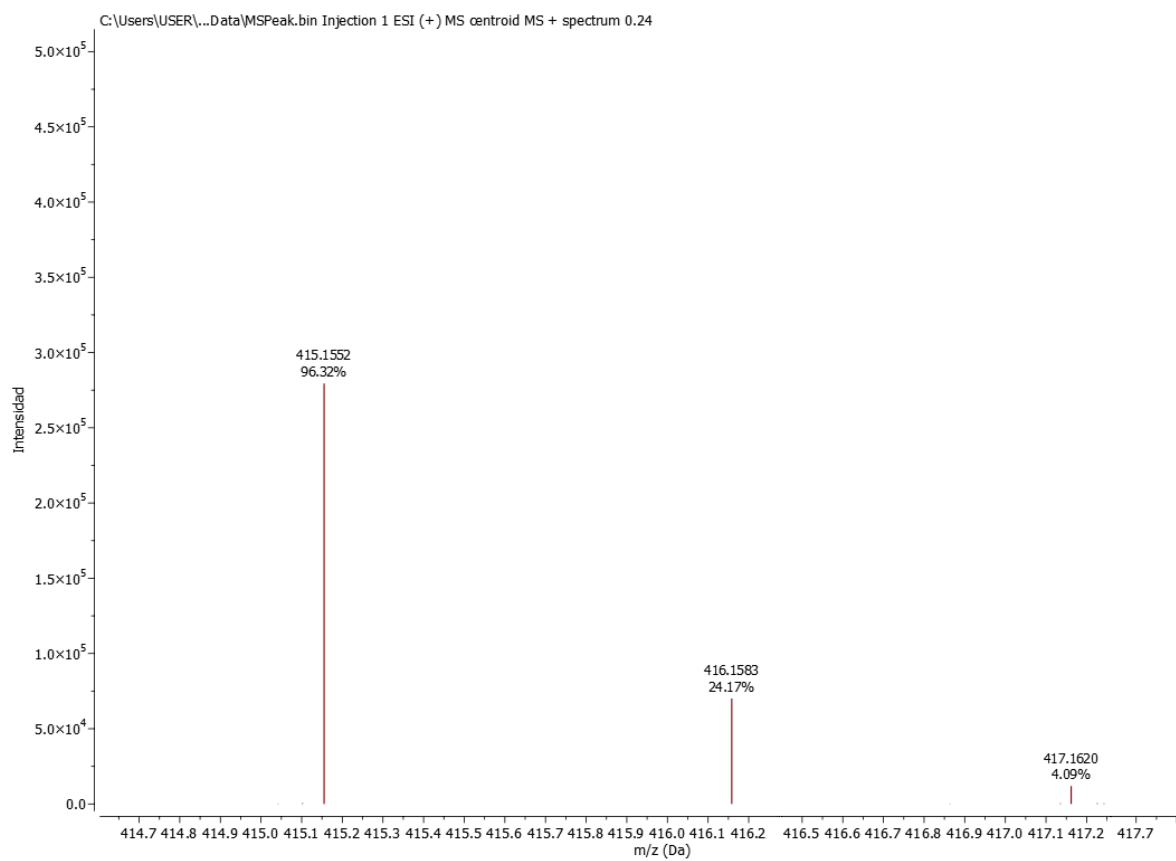

Supplement: S11. File — The physicochemical properties, spectral characterization details and copy of 1H NMR, 13C NMR and mass spectra of (E)-(4-(2,3-dimethoxystyryl)phenyl)(6-methoxybenzofuran-2-yl)methanone (6i). (PDF) [file pone.0344602.s011.pdf]
