# Supplementary material for: Novel benzofuran/pterostilbene hybrids trigger programmed cell death and impair migration in CRC cells
Source: PLoS One. 2026 Apr 13;21(4):e0344602. doi: 10.1371/journal.pone.0344602 (PMC13075696; doi:10.1371/journal.pone.0344602)
Supplement: S12. File — Spectral characterization details and copy of 1H NMR and 13C NMR of (4-(2,6-dimethoxystyryl)phenyl)(6-methoxybenzofuran-2-yl)methanone (6j). (PDF) [file pone.0344602.s012.pdf]

S12. The physicochemical properties, spectral characterization details and copy of ^{1}H NMR, ^{13}C NMR and mass spectra of *(4-(2,3-dimethoxystyryl)phenyl)(6-methoxybenzofuran-2-yl)methanone (**6j**)*.

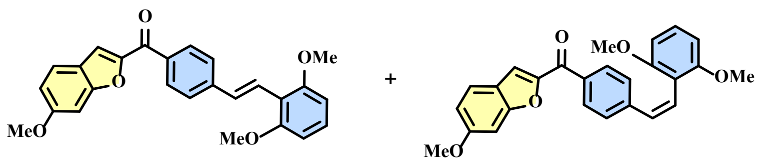

^{1}H NMR (300 MHz, $CDCl_3$) δ <u>*E*-isomer</u>: 8.02 (d, *J* = 8.4 Hz, 2H), 7.85 (d, *J* = 8.4 Hz, 2H), 7.63 (d, *J* = 16.1 Hz, 1H, $CH=CH_{trans}$), 7.55 (d, *J* = 8.7 Hz, 1H), 7.41 (s, 1H), 7.28 (d, *J* = 16.1 Hz, 1H, $CH=CH_{trans}$), 7.25 (t, *J*= 8.4 Hz, 1H), 7.10 (d, *J* = 8.7 Hz, 1H), 6.96 – 6.91 (m, 1H), 6.61 (d, *J* = 8.4 Hz, 1H), 6.54 (d, *J* = 8.4 Hz, 1H), 3.92 (s, 6H, 2 x OMe), 3.90 (s, 3H, OMe). <u>*Z*-isomer</u>: 8.02 (d, *J* = 8.4 Hz, 2H), 7.85 (d, *J* = 8.4 Hz, 2H), 7.48 (s, 1H), 7.41 (s, 1H), 7.25 (t, *J*= 8.4 Hz, 1H), 7.21 (d, *J* = 12.1 Hz, 1H, $CH=CH_{cis}$), 7.10 (d, *J* = 8.4 Hz, 1H), 7.01 – 6.96 (m, 1H), 6.78 (d, *J* = 12.1 Hz, 1H, $CH=CH_{cis}$), 6.61 (d, *J* = 8.4 Hz, 1H), 6.54 (d, *J* = 8.4 Hz, 1H), 3.88 (s, 3H, OMe), 3.63 (s, 6H, 2 x OMe). **^{13}C NMR ($CDCl_3$, 75 MHz)**: δ 183.35 (C=O), 161.13 (6-benzofuran), 158.94 (2 and 6-ring B), 157.66 (7a-benzofuran), 152.20 (2-benzofuran), 144.05 (4-ring A), 143.56 (2-ring A), 135.60 (4-ring B), 131.02 (Ar_1-**<u>C</u>**H=CH-Ar_2),129.89 (2 and 6-ring A), 129.03 (3 and 5-ring A), 127.89 (3a-benzofuran), 126.32 (Ar_1-**<u>C</u>**H=CH-Ar_2), 123.61 (4-benzofuran), 120.48 (1-ring B), 114.46 (3-benzofuran), 114.28 (5-benzofuran), 103.99 (5-ring B) and 103.86 (3-ring B), 95.70 (7-benzofuran), 55.84 (2 x OMe), 55.50 (OMe).

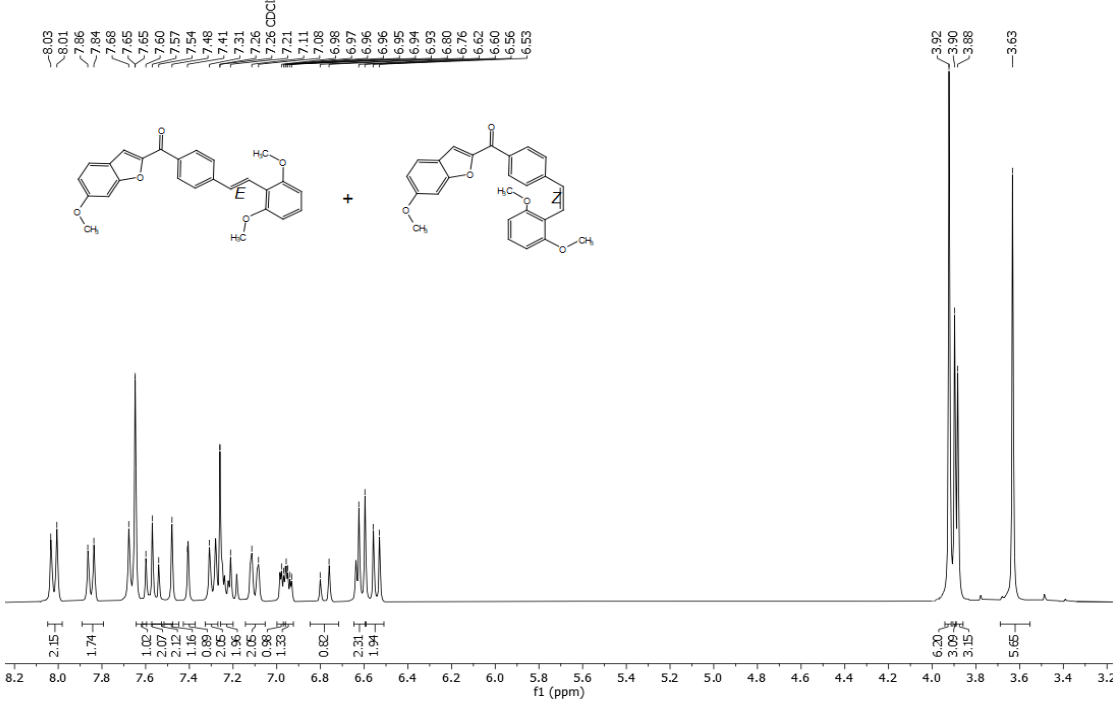

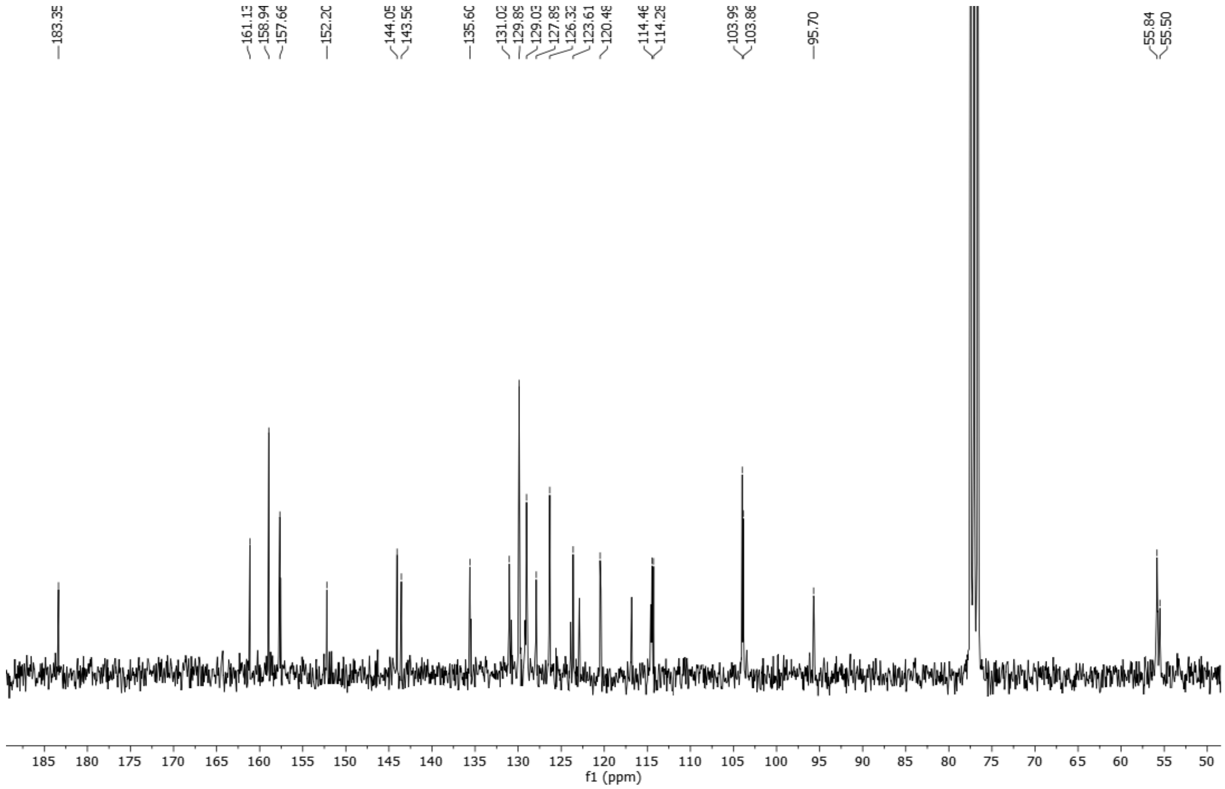
183.39
161.13
158.94
157.66
152.20
144.05
143.56
135.60
131.02
129.89
129.03
127.89
126.32
123.61
120.48
114.46
114.28
103.99
103.86
95.70
55.84
55.50
185 180 175 170 165 160 155 150 145 140 135 130 125 120 115 110 105 100 95 90 85 80 75 70 65 60 55 50
f1 (ppm)
